# The Pivotal Roles of TIA Proteins in 5′ Splice-Site Selection of *Alu* Exons and Across Evolution

**DOI:** 10.1371/journal.pgen.1000717

**Published:** 2009-11-13

**Authors:** Nurit Gal-Mark, Schraga Schwartz, Oren Ram, Eduardo Eyras, Gil Ast

**Affiliations:** 1 Department of Human Molecular Genetics and Biochemistry, Sackler Faculty of Medicine, Tel-Aviv University, Tel Aviv, Israel; 2 Computational Genomics, Universitat Pompeu Fabra, Barcelona, Spain; 3 Catalan Institution for Research and Advanced Studies, Barcelona, Spain; National Institute of Genetics, Japan

## Abstract

More than 5% of alternatively spliced internal exons in the human genome are derived from *Alu* elements in a process termed exonization. *Alus* are comprised of two homologous arms separated by an internal polypyrimidine tract (PPT). In most exonizations, splice sites are selected from within the same arm. We hypothesized that the internal PPT may prevent selection of a splice site further downstream. Here, we demonstrate that this PPT enhanced the selection of an upstream 5′ splice site (5′ss), even in the presence of a stronger 5′ss downstream. Deletion of this PPT shifted selection to the stronger downstream 5′ss. This enhancing effect depended on the strength of the downstream 5′ss, on the efficiency of base-pairing to U1 snRNA, and on the length of the PPT. This effect of the PPT was mediated by the binding of TIA proteins and was dependent on the distance between the PPT and the upstream 5′ss. A wide-scale evolutionary analysis of introns across 22 eukaryotes revealed an enrichment in PPTs within ∼20 nt downstream of the 5′ss. For most metazoans, the strength of the 5′ss inversely correlated with the presence of a downstream PPT, indicative of the functional role of the PPT. Finally, we found that the proteins that mediate this effect, TIA and U1C, and in particular their functional domains, are highly conserved across evolution. Overall, these findings expand our understanding of the role of TIA1/TIAR proteins in enhancing recognition of exons, in general, and *Alu* exons, in particular.

## Author Summary

Human genes are composed of functional regions, termed exons, separated by non-functional regions, termed introns. Intronic sequences may gradually accumulate mutations and subsequently become recognized by the splicing machinery as exons, a process termed exonization. *Alu* elements are prone to undergo exonization: more than 5% of alternatively spliced internal exons in the human genome originate from *Alu* elements. A typical *Alu* element is ∼300 nucleotides long, consisting of two arms separated by a polypyrimdine tract (PPT). Interestingly, in most cases, exonization occurs almost exclusively within either the right arm or the left, not both. Here we found that the PPT between the two arms serves as a binding site for TIA proteins and prevents the exon selection process from expanding into downstream regions. To obtain a wider overview of TIA function, we performed a cross-evolutionary analysis within 22 eukaryotes of this protein and of U1C, a protein known to interact with it, and found that functional regions of both these proteins were highly conserved. These findings highlight the pivotal role of TIA proteins in 5′ splice-site selection of *Alu* exons and exon recognition in general.

## Introduction

Alternative splicing of mRNA precursors allows the synthesis of multiple mRNA isoforms from a single primary transcript [1]–[4]. Recent analyses indicate that the majority of human genes are alternatively spliced, thus contributing significantly to human transcriptome diversity [5],[6]. Accurate removal of introns occurs by a two step reaction, conserved from yeast to mammals, that takes place in a large macromolecular complex termed the spliceosome. The spliceosome consists of five small nuclear RNAs (snRNAs; U1, U2, U4, U5 and U6) and over 200 associated proteins. Four degenerate sequences are recognized by the spliceosome: the 5′ and 3′ splice sites (5′ss and 3′ss), located at the 5′ and the 3′ end of each intron, the polypyrimidine tract (PPT) and the branch point sequence (BPS) both located upstream of the 3′ss [7].

The 5′ss consensus sequence in higher eukaryotes is comprised of nine bases that bridge the exon-intron boundary; this region is bound by a complementary region along the RNA component of the U1 snRNP. In most pre-mRNAs the base pairing of U1 snRNP and 5′ss is not perfect. Increased complementarity between U1 snRNP and the 5′ss strongly contributes to 5′ss selection [8],[9] and can shift the splicing pattern from alternative to constitutive [10],[11].

In metazoans, the four main splice signals are insufficient to allow accurate splicing. It has been estimated that these splicing signals provide, at most, half of the information required for recognition by the splicing machinery [12]. Studies of the molecular basis of splicing revealed the existence of exonic and intronic cis-acting regulatory sequences (ESRs and ISRs, respectively), which bind trans-acting factors and regulate the precise excision of introns from within eukaryotic pre-mRNA. These cis-acting elements are classified as exonic or intronic splicing enhancers and silencers, which promote or inhibit splicing, respectively. These sequences have been identified using a wide array of experimental and computational methodologies [13]–[21] and interact in a complex manner to allow precise splicing [22]. Aberrant regulation of splicing is linked with a wide array of disease states, including cancer [23]–[25].

The ESRs and ISRs are recognized by trans-splicing factors, which usually contain one or more RNA binding domains as well as additional domains that are essential for recruitment of the splicing apparatus and for splice site pairing. The TIA1 (T-cell intracellular antigen 1) and TIAR (TIA1 related protein or TIAL) proteins are examples for two such splicing factors. These proteins contain an RNA-recognition motif (RRM) known as RRM2 that specifically binds U-rich RNA sequences within introns [26]. The proteins are also characterized by two additional RRMs and a glutamine rich carboxyl terminal region [26]–[28]. Binding of TIA1 protein to uridine-rich sequences downstream of weak 5′splice sites helps to recruit U1 snRNP to the 5′ss through protein-protein interactions involving the glutamine rich domain of TIA1 and the U1-specific protein U1C [29]–[32]. TIAR can also recruit U6 snRNP to a pseudo-5′ss that is followed by a U-rich sequence located within a 200-bp element regulating alternative splicing of the calcitonin/CGRP gene [33]. Because of their affinity for U-rich sequences, TIA proteins are often antagonized by the pyrimidine tract binding protein (PTB), a general repressor of exon inclusion [34]–[37]. The functions of TIA1/TIAR proteins and homologues have been demonstrated in several model organisms. In yeast, NAM8, PUB1 and NGR1 are related to the TIA proteins and have similar domain organizations. NAM8 stabilizes commitment complexes and facilitates weak 5′ss recognition by interacting with non-conserved sequences downstream of the 5′ss [38],[39]. The mouse homologs of the TIA proteins were shown to be functional as well [29],[40]. In Drosophila, Rox8 was shown to be the functional homolog, based on RNA interference experiments [41], and in plants, the related proteins UBP1 and RBP45 were shown to interact with U-rich elements and enhance splicing in [42]–[44]. Much less is known about the TIA homologs among other eukaryotes.

More than 5% of alternatively spliced internal exons in the human genome are derived from *Alu* elements. Throughout the course of evolution, some intronic *Alu*s have accumulated mutations that led the splicing machinery to select them as internal exons, a process called “exonization” [10], [45]–[47]. The majority of *Alu*-derived exons are alternatively spliced [46],[48] allowing the enrichment of the human transcriptome with new isoforms without compromising its original repertoire [49]. *Alu*s originated from the 7SL RNA gene [50]. They belong to the short interspersed elements (SINE) family of repetitive elements and are unique to primates [51],[52]. More than one million copies are dispersed throughout the human genome with a majority located in introns [46]. A typical *Alu* element is ∼300 nucleotides long, consisting of two arms (left and right) joined by an A-rich linker and followed by a poly(A) tail. The right and left arms are highly similar, sharing ∼80% of their sequence. Both arms contain potential splicing signals and both can undergo exonization, although exonizations tend to occur from the right arm [53]–[55].

When *Alus* insert into introns in the antisense orientation (relative to the coding sequence), the poly(A) tail becomes a poly(U) in the mRNA precursor and thus can serve as a PPT. This PPT presumably leads the splicing machinery to select a downstream AG as the 3′ss and a further downstream GT or GC sequence as the 5′ss [55]. Exonizations can occur either from the right *Alu* arm or from the left arm. In the first case, both the 3′ss and the 5′ss are selected from the right arm, whereas in the latter both signals are selected within the left arm. Only few cases were known to us in which the 3′ss occurs in one arm, and the 5′ss in the other, although there are many cases in which potential splicing signals are present [46],[56]. We thus hypothesized that the second PPT sequence, located within the *Alu* element and separating the two *Alu* arms from each other, limits splice site selection and causes both splicing signals to be selected from within the same arm.

To evaluate this hypothesis, we created an *Alu*-based model system of two competing 5′ss separated by a PPT. The PPT in this system is not the classical PPT located upstream of the 3′ss, but rather is a pyrimidine-rich stretch located downstream of the 5′ss. We showed that the presence of the PPT sequence led to selection of the upstream 5′ss even in the presence of a stronger 5′ss downstream. Deletion of the PPT sequence shifted selection to the stronger 5′ss. We show that this enhancing effect depended on the strength of the downstream 5′ss and the efficiency of base pairing to U1 snRNA. PPTs of 3-to-9 nucleotides modulated different levels of 5′ss usage. We also show that this enhancing effect is mediated by the binding of TIA proteins to the *Alu* PPT and that the function of these proteins is distance-dependent. To obtain a wide-scale overview on the evolution of the TIA proteins and their binding sites, we analyzed over 1 million introns from 22 eukaryotes and found that throughout eukaryotic evolution there has been an increased tendency for PPTs to occur within ∼20 nt downstream of the 5′ss. Among most metazoans, the strength of the 5′ss inversely correlates with the presence of a downstream PPT, indicating the functional importance of this signal. Finally, we searched for TIA homologs across evolution and found that functional regions of these proteins are highly conserved. Taken together, these findings indicate that throughout eukaryotic evolution, the TIA proteins have served as key players that have helped shape introns and that these proteins also mediate the formation of new exons, as in the context of *Alu* exonizations.

## Methods

### Plasmid construction

The ADAR2 minigene, containing the human genomic sequence of exons 7, 8 and 9 (2.2kb), was previously cloned [45]. The PCR products were restriction digested and inserted between the *KpnI*/*BglII* sites in the pEGFP-C3 plasmid (Clontech), which contains the coding sequence for Green Fluorescent Protein (GFP). The 350-nt intronic sequence originating from intron 11 of the IMP gene was amplified by PCR using 5′ phosphorylated primers and inserted downstream of the PPT sequence of the intronic left arm of the *Alu* element. For RNA pull-down assays, three fragments containing the 5′ss of the *Alu* exon and the PPT downstream of it were amplified by PCR from WT, ΔPPT and rep_PPT minigenes and cloned into the BamHI/EcoRI sites of pBluescript KS+. The TIA1b and TIARb cDNAs (kind gifts from Juan Valcárcel) were cloned into the pEGFP-C1 vector and the U1 gene was cloned into the pCR vector. For the sequences of the ADAR minigene insert and pBluescript KS+ inserts see Text S1.

### Minigene mutagenesis

Site-directed mutagenesis was carried out to introduce mutations into the ADAR2 and U1 minigenes by PCR using oligonucleotide primers containing the desired mutations. Mutations creating deletions in wild-type minigenes were performed by PCR using 5′ phosphorylated primers flanking the sequence to be deleted (see Supplementary Table 1 in Text S1 for list of primers). PCR was performed using *PfuTurbo* DNA polymerase (Stratagene) with an elongation time corresponding to 2 min for each kb. The PCR products were treated with *DpnI* (20 U, New England BioLabs) at 37°C for 1 h. Plasmid mutants were ligated using T4 DNA Ligase (New England BioLabs) at 37°C for 2 h. The mutant DNA was transformed into *E. coli* XL1-competent cells. DNA was extracted from selected colonies by mini-prep extraction (Promega). All plasmid sequences were confirmed by sequencing.

### Transfection, RNA isolation, and RT–PCR amplification

293T cells were cultured in Dulbecco's Modification of Eagle medium, supplemented with 4.5 g/mL glucose (Biological Industries, Inc.), 10% fetal calf serum (FCS), 100 U/mL penicillin, 0.1 mg/mL streptomycin and 1 U/mL nystatin (Biological Industries, Inc.). Cells were cultured in 6-well plates under standard conditions at 37°C in 5% CO_2_. Cells were grown to 50% confluence and transfection was performed using 3 µL *Trans*IT LT1 (Mirus) with 1 µg of plasmid DNA. RNA was isolated and harvested after 48 h. Total RNA was extracted using Trizol Reagent (Sigma), followed by treatment with 1 U RNase-free DNase (Ambion). Reverse transcription (RT) was preformed for 1 h at 42°C using an oligo dT reverse primer and 2 U reverse transcriptase of avian myeloblastosisvirus (AMV, Roche). The spliced cDNA products derived from the expressed minigenes were detected by PCR using an ADAR2 exon 7 forward primer ( ^5′^CCCAAGCTTTTGTATGTGGTCTTTCTGTTCTGAAG^3′^ ) and a pEGFP-specific reverse primer ( ^5′^CGCTTCTAACATTCCTATCCAAGCGT^3′^ ). Amplification was performed for 28 cycles to maintain a linear relationship between the input RNA and signal [18]. Each cycle consisted of 30 sec at 94°C, 45 sec at 61°C and 1.5 min at 72°C. The RT-PCR products were separated on a 2% agarose gel and confirmed by sequencing. The relative ratios of RNA products using 5′ssA or 5′ssB were measured using ImageJ software (http://rsb.info.nih.gov/ij/index.html), as we previously established that ImageJ quantification for ADAR2 RT-PCR products correlates with real-time RT-PCR quantification produced by the Roche LightCycler PCR and detection system [45]. Semi-quantitative RT-PCR of three independent biological replicates of three ADAR minigene mutants revealed standard deviations of 0.6% to 5.3% of the relative ratios of RNA products.

### RNA pull-down assays

Linearized pBluescript KS+ plasmids were used as templates for the synthesis of biotinylated RNAs by using T7 RNA polymerase (Promega) and biotinylated-16-UTP (Roche) following manufacture recommendations. Total cell extract from 1 mg of HeLa cells was incubated with 1 µg of biotin-labeled RNA and rotated for 4 h at 4°C in binding buffer containing 10 mM HEPES, pH 7.5, 40 mM KCl, 3 mM MgCl_2_, 5% glycerol, supplemented with 40 units of RNasin (Promega) and 5 mg/ml heparin (Sigma). The biotin-labeled RNA was isolated using streptavidin-conjugated beads (Fluka) and was washed with binding buffer for four times. The presence of TIA1/TIAR in the pull-down pellet was verified by western blot analysis as described below.

### Western blotting

Lysis buffer (50 mM Tris at pH 7.5, 1% NP40, 150 mM NaCl, 0.1% SDS, 0.5% deoxycholic acid, protease inhibitor cocktail and phosphatase inhibitor cocktails I and II; Sigma) was used for protein extraction. Lysates were centrifuged for 30 min at 14,000 rpm at 4°C. Total protein concentrations were measured using BioRad Protein Assay (Bio-Rad). Proteins were separated in 12% SDS-polyacrylamide gel electrophoresis (SDS-PAGE) and then electroblotted onto a Protran membrane (Schleicher and Schuell). The membranes were probed with anti-TIA1 (C-20, Santa Cruz Biotechnology), anti-TIAR (C-18, Santa Cruz Biotechnology), anti-HSC70 (B6, Santa Cruz Biotechnology), anti-GFP (MBL) or anti-α-tubulin (Sigma), followed by the appropriate secondary antibody. Immunoblots were visualized by enhanced chemiluminescence (Lumi-Light Western Blotting Substrate; Roche) and exposure to X-ray film.

### Search for exonization events within *Alu* elements

To examine the prevalence of *Alu* exons with a 3′ss selected within the right arm and a 5′ss within the left arm, we began by querying the TranspoGene webserver [57] for cases of exons overlapping *Alu* elements in the antisense orientation that were supported by at least one EST. This query yielded 744 such exons. Since we were interested only in cases in which both the 5′ss and the 3′ss occurred within the *Alu* sequence, we next filtered out all cases in which either of these signals occurred outside of the *Alu* sequence; this yielded 548 sequences. To map the 3′ss and the 5′ss of each exonization event to either the left or the right arm performed pairwise alignments between each *Alu* and the *Alu*-Jo consensus sequence based on the Needleman-Wunsch algorithm for global alignment [58].

### Identification of PPTs downstream of the 5′ss

To identify PPTs, we used the algorithm we previously developed for identifying polypyrimidine tracts that is described in detail in [54]. We set a minimum score threshold of 6, which dictates that a PPT sequence must consist of at least six consecutive pyrimidines. Notably, the identified stretch may also be longer and may contain non-pyrimidines as long as the overall enrichment score is ≥6.

For each intron of each organism, we first masked the 30 terminal nucleotides and then searched for pyrimidine-rich stretches within the 300 first nucleotides of the intron or within the entire remaining stretch of the intron in cases of introns shorter than 330 nucleotides. The 30 terminal nucleotides were masked in order to avoid contamination by PPT at the 3′ end of the intron. To derive the plots indicating the presence of PPTs for each organism, we summarized for each of the first 100 intronic positions the number of PPTs covering that position and divided this number by the number of introns reaching that position.

### 5′ss scoring

The 5′ss of all introns were scored based on their adherence to a position-specific scoring matrix (PSSM) for the 5′ss consensus for each organism. The 5′ss was defined as 12 positions as in [54], including four exonic and eight intronic positions. The 5′ss score was calculated as:
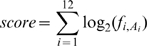
where A is the sequence to be scored and f_i,Ai_ is the PSSM frequency at position i of the *i*th nucleotide in sequence A.

### Examination of PPTs flanking alternative 5′ events

A dataset of 3634 alternative 5′ss events, based on the AltSplice track in University of California Santa Cruz (UCSC) Genome Browser (http://genome.ucsc.edu/) was compiled. We discarded all events in which the distance between the two alternative 5′ss was less than 12 nt, in order to allow the presence of a PPT. This left 2,296 alternative 5′ss events. PPTs within the first 25 nt (or less) downstream of each of the two competing 5′ss were found as described above.

### Identification of TIA homologs

We compiled a set of 684 known splicing factors with RNA binding domains from multiple species. We then grouped these proteins into 38 families (see Supplementary Table 3 in Text S1). We grouped known TIA1/TIAR and polyuridylate binding proteins (PUB1) proteins into the same family and known NAM8 and NGR1 proteins into a different family. For each family we built a hidden Markov model (HMM) for each of the RNA binding domains (RRM or KH-type) using Hmmer [59] (http://hmmer.janelia.org). We considered as candidate homologs those proteins that had collinear hits for a multidomain protein in the right order or a single hit for a single domain protein. For each of the sets of homologous RRMs we built a maximum parsimony tree using the close-neighbour-interchange algorithm with search level 3. The initial trees were obtained with random addition of sequences using 10 replicates. A candidate protein was labeled as an ortholog of a known protein if its RRMs grouped consistently in the trees with most of the known RRMs (see Dataset S1). Multiple alignments were built using t-coffee [60] and phylogenetic analyses were performed with MEGA4 [61].

In order to establish the conservation between proteins or between domains we used two measures: the average pairwise identity and the multiple alignment conservation score. For the average pairwise identity we calculated, for each pair, the proportion of identical amino acids over the gapless positions and averaged over all pairs in the multiple sequence alignment (MSA). To calculate the conservation score (MSA score), we first calculated the score for each gapless column of the MSA by determining the proportion of amino acid pairs *M* in the column that were identical:
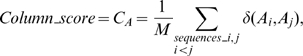
where *δ(A_i_,A_j_)* is 1 if *A_i_ = A_j_* and 0 otherwise. The MSA score was then computed as the average of the column scores over all the gapless columns *N*:
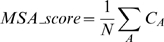



## Results

### The *Alu* left arm PPT enhances 5′ss selection in the right arm *Alu* exon

The left and right arms of *Alu* elements are highly similar and both contain potential splice sites [53]. However, in most cases exonization occurs almost exclusively within either the right arm or the left, but not both. We were interested in determining how often exonizations overlapped both arms. We compiled a dataset of *Alus* in the antisense orientation involved in exonization events, based on a TranspoGene query [57]. We then used pairwise alignments against an *Alu* consensus sequence to map each signal to the right or the left arm. Of 548 cases of exonization events within *Alu* elements, 405 (74%) occurred from within the right arm only, 114 (21%) occurred from within the left arm only, and in only 29 (5%) was the 3′ss selected from within the right arm and the 5′ss from within the left.

**Figure 1:**
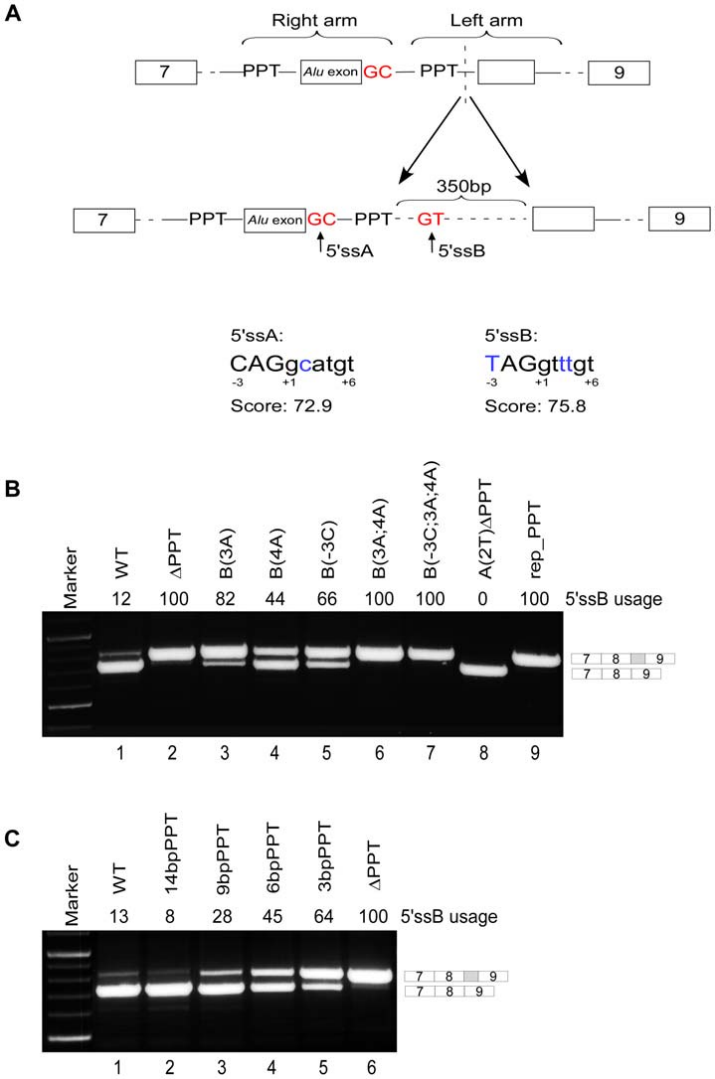
The effect of a PPT located between two putative 5′ss on 5′ss selection. (A) Illustration of the original (upper part) and modified (lower part) ADAR2 minigenes (referred as ADAR WT in the manuscript). The original ADAR2 minigene contains exons 7 to 9 of the human ADAR2 gene (exons are indicated by boxes), along with the introns between them. Exon 8 is an *Alu* exon that originated from the right arm of the *Alu* element and is alternatively spliced (the right and left arms of the *Alu* element are marked by horizontal brackets). A 350-bp sequence was inserted downstream of the left arm PPT of the *Alu* element. The 5′ss of the *Alu* exon is defined as 5′ssA and the 5′ss generated within the 350-bp insert is defined as 5′ssB. 5′ssA and 5′ssB are indicated by arrows and their sequences and Shapiro and Senapathy scores (http://ast.bioinfo.tau.ac.il/SpliceSiteFrame.htm) are shown. The positions subjected to mutations are marked in blue. (B) ADAR2 minigenes containing the indicated mutants were transfected into 293T cells. Total cytoplasmic RNA was extracted and splicing products were separated in 2% agarose gel after RT-PCR. Lane 1, splicing products of wild-type ADAR2; lane 2, splicing of the product of a minigene with deletion of the PPT sequence downstream to 5′ssA; lanes 3–8, splicing products of mutants that strengthened 5′ss A or B; lane 9, splicing products of a minigene with the 14-bp PPT sequence replaced with a sequence that does not contain splicing regulatory elements. The PCR products were identified by sequencing and the two minigene mRNA isoforms are shown on the right. The numbers above the lanes indicate the 5′ssB usage. (C) Splicing assays were performed as described in (B). Lane 1, splicing products of wild-type ADAR2. Lanes 3–6, effects of shortening of the PPT between the two competing 5′ss.

In light of our finding that *Alu* exonization events do not tend to cross the border between the two arms, we hypothesized that the PPT sequence separating the two *Alu* arms prevents exonization into downstream sequences. To examine this hypothesis, we used a modified version of the ADAR2 minigene as a model system. The original ADAR2 minigene contains exons 7 to 9 of the human ADAR2 gene, along with the introns between them. Exon 8 is an *Alu* exon that originated from the right arm of the *Alu* element and is alternatively spliced. In order to investigate the effect of the PPT in isolation of the pseudo-exon effect of the left arm [53], we began by inserting a 350-nt sequence between the PPT and the potential 3′ss of the left arm (Figure 1A). By separating the right arm from the left by 350nt, we eliminated the effect of the intronic arm on the *Alu* exon, thus shifting splicing of the *Alu* exon from alternative to constitutive splicing [53]. We next generated a 5′ss 68 nucleotides downstream of the PPT of the intronic left arm (5′ssB in Figure 1A). This 5′ss is stronger in terms of Senapathy score (http://ast.bioinfo.tau.ac.il/SpliceSiteFrame.htm) than the 5′ss of the *Alu* exon (5′ssA in Figure 1A). Thus, this system contains two potential 5′ss separated by a PPT sequence (Figure 1A); we will henceforth refer to this minigene as ADAR WT, and to the PPT following the *Alu* exon (originating from the left arm) as PPT.

The minigene was transfected into 293T cells, total cytoplasmic RNA was extracted after 48 hours and 5′ss selection was examined by RT-PCR analysis using primers specific to the minigene mRNA. Although 5′ssA is weaker than site B, it was almost exclusively selected (Figure 1B, lane 1). However, when the PPT sequence was deleted or replaced by a sequence that did not contain any splicing regulatory elements (see sequence in Supplementary Methods in Text S1) there was a shift in 5′ss selection from site A to site B (Figure 1B, lanes 2 and 9, respectively). These results indicated that the PPT enhances selection of a weaker upstream 5′ss in the presence of a stronger 5′ss downstream.

To determine whether this enhancing effect of the PPT was dependent on the strengths of the 5′ss, we made mutations in 5′ssB to strengthen it over a Senapathy score range of 79.87 to 100. Specifically, we inserted different combinations of T→A mutations in positions 3 and 4, and a T→C mutation in position −3. As site B was strengthened, there was a gradual shift towards selection of this site despite the presence of the PPT sequence (Figure 1B, compare lane 1 to lanes 3–7). Strengthening of 5′ssA in combination with a deletion of the PPT sequence resulted in its constitutive selection (Figure 1B, lane 8). These results imply that there is a delicate interplay between the PPT and the strengths of the splice sites flanking it. The presence of a PPT sequence enables selection of a weak 5′ss upstream, but only if the downstream 5′ss is weaker than a certain level. Once the competing 5′ss is strong enough, it is selected despite the presence of the PPT.

To determine the length of the PPT required for efficient selection of site A, we shortened the 14-nt PPT sequence separating site A from site B to nine, six and three consecutive uridines. Shortening the PPT resulted in a shift from site A to B (compare Figure 1C, lane 1 to lanes 3–5). When the two adenosine bases within the PPT sequence in the WT minigene (see minigene sequence in Supplementary Methods in Text S1) were replaced with uridines to obtain a PPT of 14 consecutive uridines, there was little change in 5′ss selection compared to the WT (Figure 1C, lane 2). These results indicated that a PPT with at least nine consecutive uridines results in maximal selection of 5′ssA.

**Figure 2:**
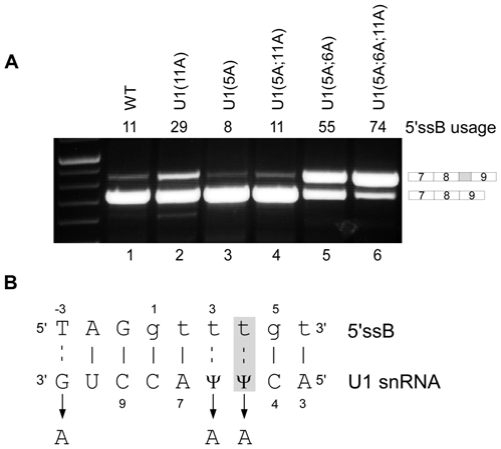
U1 snRNA affects selection of 5′ssB. (A) A plasmid containing the U1 snRNA cDNA with the indicated mutations was co-transfected with the ADAR2 WT minigene into 293T cells. The U1 snRNA mutations are numbered according to the positions indicated in (B). Splicing assays were performed as described in Figure 1. Lane 1, splicing products of wild-type ADAR2; lanes 2–6, splicing products of ADAR2 minigene co-transfected with U1 mutants. The two minigene mRNA isoforms are shown on the right. The numbers above the lanes indicate the 5′ssB usage. (B) Schematic illustration of the base pairing between 5′ssB and U1. Positions of 5′ssB and U1 are numbered forward and reverse, respectively. Mutations inserted in positions 5, 6, and 11 are indicated by arrows. The mutation in position 5 of the U1 snRNA (marked in gray) is complementary to both 5′ssA and 5′ssB. Watson-Crick and non-Watson-Crick base pairing are marked by solid or dashed lines, respectively.

We then set out to examine whether the competition between the two putative 5′ss is mediated through the binding to U1 snRNA. 293T cells were co-transfected with the ADAR WT minigene and with a U1 gene containing mutations to enhance complementarity to site B (Figure 2A). A schematic illustration of the base pairing between site B and U1 is presented in Figure 2B. Mutations were made at positions 5, 6 and 11 of U1 snRNA to improve its base pairing to 5′ssB (these U1 snRNA mutations are complementary to positions 4, 3 and −3 in 5′ssB, respectively). Improving the binding of U1 snRNA to 5′ssB by insertion of all three mutations enhanced its selection (Figure 2A, compare lane 1 to lane 6), indicating that complementarity to U1 snRNA is critical to 5′ss selection in this competitive situation. Notably, an individual mutation at position 5 of U1 snRNA or the combination of mutations in positions 5 and 11 did not improve base pairing of U1 snRNA to 5′ssB. This is presumably explained by the fact that the mutation at position 5 enhances the ability of U1 snRNA to base pair not only with 5′ssB but also with 5′ssA (Figure 2B). The reciprocal experiment, in which a U1 snRNA was designed with complementarity to 5′ssA, caused activation of a cryptic intronic site that resembles 5′ssA (data not shown).

### TIA proteins enhance 5′ssA selection and their function is distance-dependent

**Figure 3:**
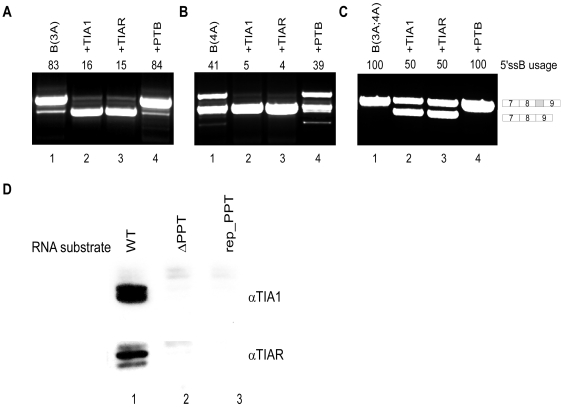
Effects of TIA1, TIAR, and PTB on alternative 5′ss selection. Splicing assays were performed as described in Figure 1. (A) Lane 1, splicing products of B(3A) ADAR2 mutant minigene in which 5′ssB was strengthened at position 3 by mutation from T to A. Lanes 2–4, the B(3A) mutant was co-transfected with plasmids that expressed TIA1, TIAR and PTB, respectively. (B) Lane 1, splicing products of B(4A) ADAR2 mutant minigene in which 5′ssB was strengthened at position 4 by mutation from T to A. Lanes 2–4, co-transfection of B(4A) with TIA1, TIAR and PTB, respectively. (C) Lane 1, splicing products of B(3A;4A) ADAR2 mutant minigene in which 5′ssB was mutated at both positions 3 and 4. Lanes 2–4, co-transfection of B(3A;4A) with TIA1, TIAR, and PTB, respectively. The two minigene mRNA isoforms are shown on the right. The numbers above the lanes indicate 5′ssB usage. (D) TIA1 and TIAR bind to the PPT downstream of the *Alu* exon. A pull-down assay using biotinylated RNA probes was performed following incubation with HeLa total cell extract. After RNA pull-down using streptavidin-conjugated beads, the samples were loaded onto a 12% SDS-polyacrylamide gel and were analyzed by western blot for TIA1 and TIAR.

It has been previously shown that TIA proteins (TIA1 and TIAR) activate weak 5′ss that are located upstream of U-rich sequences [29]–[33]. To test whether the enhancing effect of the PPT sequence on the selection of the weak 5′ssA is mediated by the binding of TIA1/TIAR, we transfected 293T cells with three mutant minigenes that contained 5′ssB of different strengths and thus exhibited different levels of site B selection. For variants B(3A4A), B(3A) and B(4A), 5′ssB was selected in 100%, 82% and 44% of the transcripts, respectively (Figure 1B). We also co-transfected the cells with vectors containing TIA1 and TIAR. In addition, we co-transfected the cells with a vector containing the PTB cDNA, which is also known to bind pyrimidine rich sequences [35]. As shown in Figure 3, co-transfection of the indicated mutants with TIA1 and TIAR cDNA induced a shift of splicing towards use of 5′ssA. Western blot analysis revealed that both proteins were expressed at the same level (see Supplementary Figure 1 in Text S1). However, co-transfection of the same mutants with PTB did not affect the splicing pattern of any of these ADAR mutants. We subsequently depleted levels of the TIA proteins via siRNA experiments. In these experiments we did not observe a shift in the 5′ss selection, which may be explained either by functionality of the residual levels following depletion or by involvement of additional factors (data not shown).

We then determined whether TIA1 and TIAR could bind to the PPT sequence downstream of 5′ssA. Three fragments containing the 5′ss of the *Alu* exon and the downstream PPT were amplified by PCR from the WT ADAR minigene and from the mutant minigenes in which the PPT sequence was deleted or replaced (ΔPPT and rep_PPT minigenes, respectively, see Figure 1B). The fragments were cloned into pBluescript KS+ plasmids (see insert sequences in Supplementary Methods in Text S1) and in vitro transcription using T7 RNA polymerase and biotinylated-16-UTP was performed. Biotinylated transcripts were incubated with HeLa extracts, isolated by streptavidin-conjugated beads and TIA1 and TIAR was detected using western blot analysis. Our results indicate that TIA proteins strongly interact with the RNA transcript corresponding to 5′ssA and the PPT sequence downstream of it: The anti-TIAR and anti-TIA1 antibodies detected double bands at 40 and 44 kD, corresponding to two different isoforms of TIAR and TIA1, respectively [62], when the WT biotinylated RNA was used (Figure 3D, lane 1). The TIA1 and TIAR bands were completely absent when the PPT sequence was deleted or replaced (Figure 3D, lanes 2 and 3, respectively).

**Figure 4:**
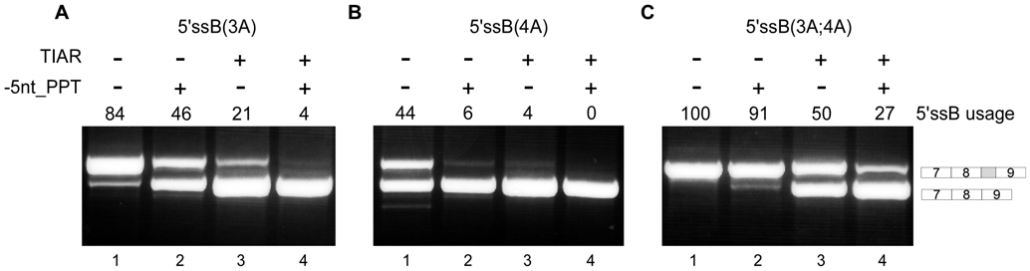
The effect of TIAR on 5′ss selection in the ADAR2 minigene is distance-dependent. Splicing assays were performed as described in Figure 1. (A) Lane 1, splicing products of B(3A) ADAR2 mutant minigene. Lane 2, splicing products of the B(3A) ADAR2 mutant minigene after deletion of five nt between the 5′ssA and the PPT. Lane 3, as in lane 1 but with co-transfection of TIAR. Lane 4, as in lane 2 except with co-transfection of TIAR. (B) Splicing analysis of B(4A) ADAR2 mutant minigene in experiments analogous to that described in (A). (C) Splicing analysis of B(3A;4A) ADAR2 mutant minigene in experiments analogous to that described in (A).

Previous studies have experimentally demonstrated that the splicing-enhancing function of U-rich sequences is observed when they are located immediately downstream from the activated 5′ss [29],[30]. In our model system the PPT is located 18 nt from 5′ssA yet still enhances selection of 5′ssA. To examine whether positioning of the PPT sequence in closer proximity to 5′ssA would enhance its selection further, we deleted five nucleotides from the 18-nt sequence separating the PPT from 5′ssA (indicated as −5nt_PPT in Figure 4), using the B(3A), B(4A) and B(3A4A) mutants. Deletion of five nucleotides from the 18-nt sequence separating the PPT from 5′ssA also shortened the distance between 5′ssA and B. Interestingly, deletion of five nucleotides resulted in a shift of splicing from 5′ssB to 5′ssA (Figure 4, compare lanes 1 and 2 in each panel). Deletion of five or ten nucleotides of the sequence lying between the PPT sequence and 5′ssA had the same effect on 5′ss usage in mutants B(3A), B(4A) and B(3A4A) ADAR mutants (Supplementary Figure 2A in Text S1) and a negligible effect on the splicing pattern of the ADAR WT minigene (Supplementary Figure 2B in Text S1). Furthermore, deletion of five nucleotides from the sequence separating the PPT sequence and 5′ssA in the presence of TIAR resulted in predominant selection of 5′ssA (Figure 4, compare lanes 1 and 4 in each panel). Taken together, these results demonstrate that the enhancing effect of TIAR on the selection of a weak 5′ss decreases with distance.

### PPTs downstream of the 5′ss are enriched throughout eukaryotic evolution

**Figure 5:**
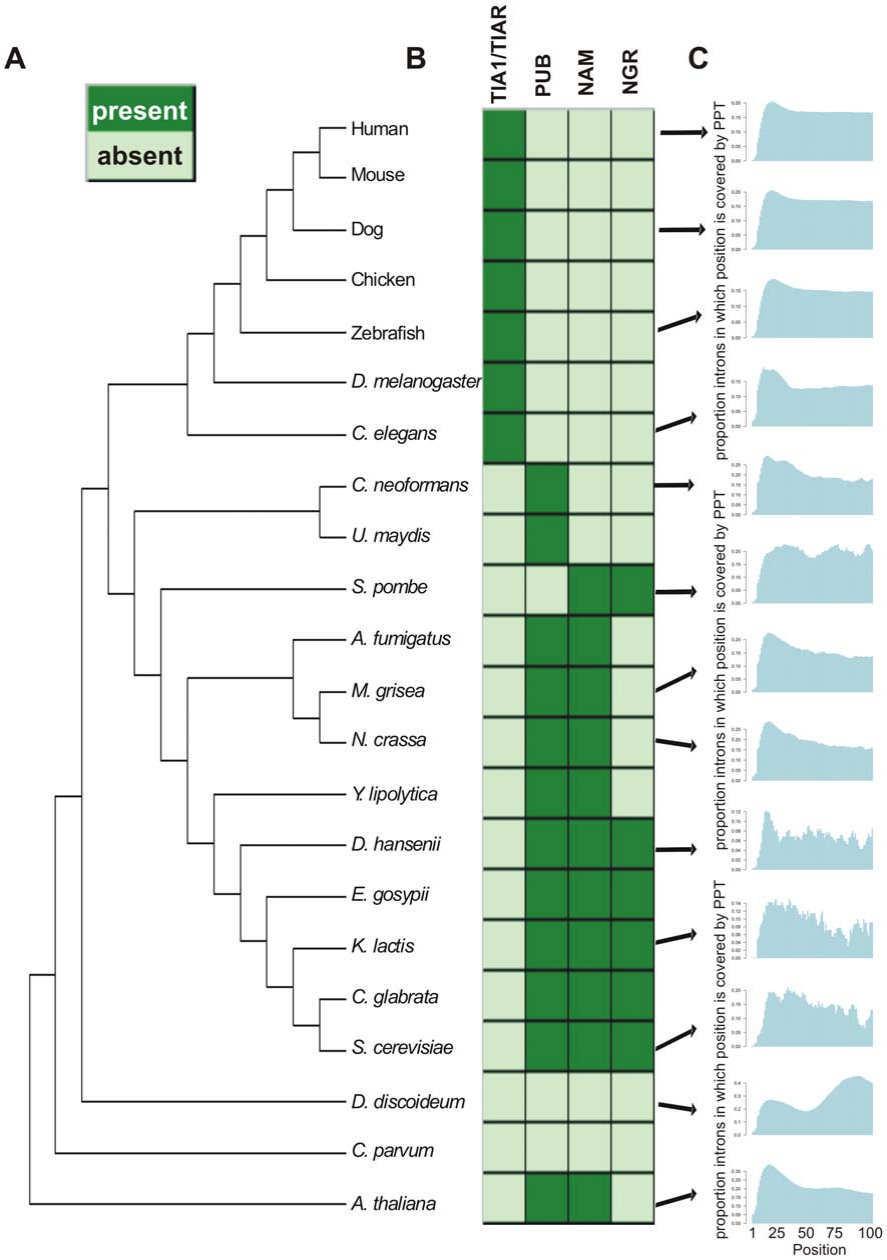
Evolutionary analysis of TIA binding sites and proteins across 22 organisms. (A) Phylogenetic tree of the 22 organisms analyzed in this study. (B) A heat chart is presented depicting the presence/absence of TIA1/TIAR, PUB1, NAM8, and NGR1 homologs. Presence and absence of a homolog is indicated by dark or light green, respectively. (C) Plots portraying the prevalence of PPTs within the first 100 nt of the introns. The y-axis indicates the proportion of introns in which a given position is covered by a PPT, following normalization to intron length. Results for additional organisms are shown in Supplementary Figure 3A in Text S1.

Our analyses thus far indicated that in our *Alu* model, the PPT between the two *Alu* arms was bound by TIA proteins and enhanced selection of the weaker, upstream 5′ss. We were thus interested in understanding the impact of TIA proteins across evolution. Specifically, we focused on three components: the TIA binding sites on pre-mRNA, the TIA proteins, and the protein U1C, which serves as a link between the TIA proteins and the 5′ss [31]. It has been previously shown that the 5′ end of human introns are enriched in U-rich tracts [63], but other organisms have not been analyzed for this phenomenon. To determine how wide-spread this enrichment is, we determined the prevalence of PPTs downstream of the 5′ss in a dataset of over 1 million introns from 22 organisms spanning all four major eukaryotic kingdoms: plants, protozoans, fungi and metazoans (Figure 5A). Strikingly, we found an enrichment of PPTs downstream of 5′ss in almost all organisms in the dataset (Figure 5C and Supplementary Figure 3A in Text S1). PPTs were found in ∼20 to 40% of the introns and, in most cases, the center of the PPT was located between positions 15 and 25 downstream of the 5′ss (see Supplementary Table 2 in Text S1). The mean lengths of the PPTs ranged from 10 to 14 nucleotides depending on the organism (Supplementary Table 2 in Text S1). Notably, among several fungi, including *S. pombe*, *U. maydis*, *Y. lipolytica* and *E. gossypi*, as well as in the protozoan *C. parvum*, the pyrimidine-rich peaks were less pronounced. This may be indicative either of functional aspects, or may result from the fact that these organisms have fewer introns, making our measurements in these organisms less reliable.

### Anti-correlation between 5′ss strength and prevalence of PPT downstream of the 5′ss

**Figure 6:**
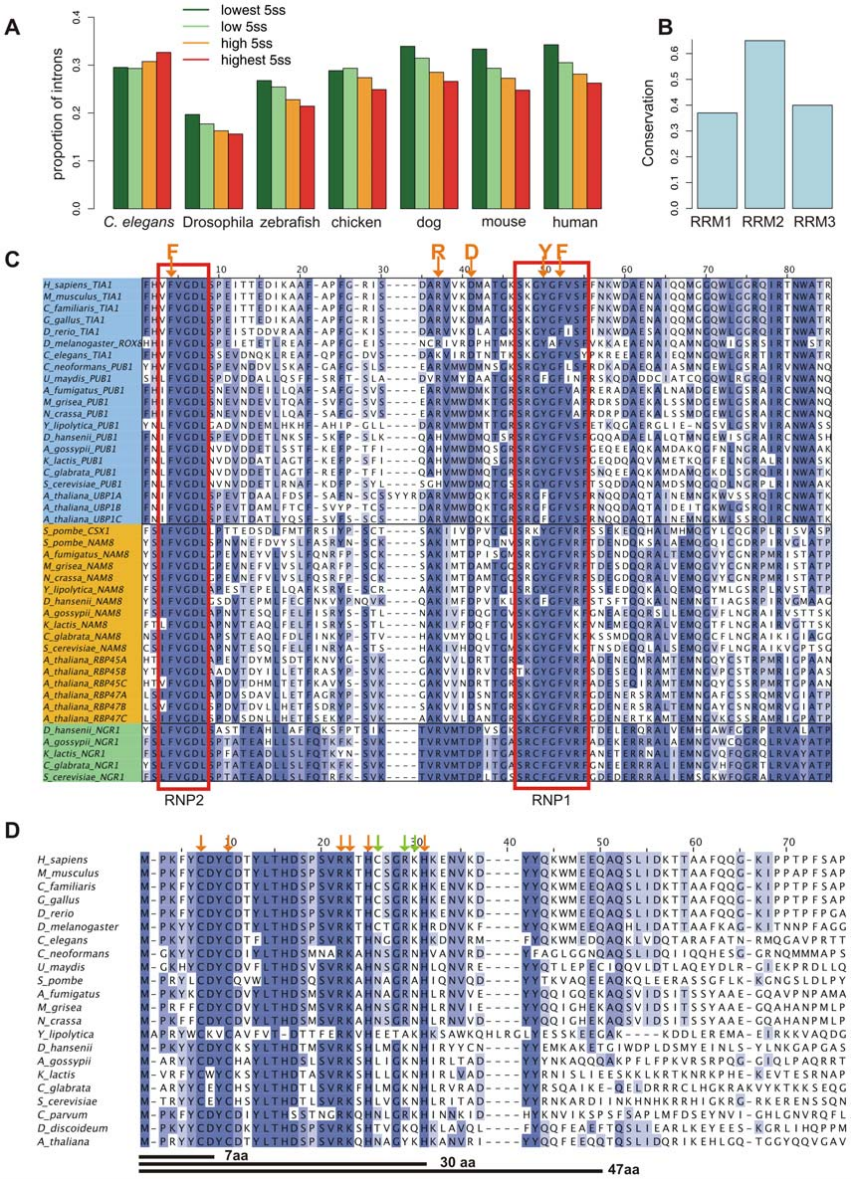
Evolutionary analysis of TIA binding sites and proteins. (A) Proportion of introns with a PPT within 20 nt from the 5′ss, as a function of 5′ss strength, among the indicated metazoans. Results for additional organisms are shown in Supplementary Figure 3B in Text S1. (B) Conservation of RRMs among TIA homologs. Multiple alignment conservation scores were calculated as described in Methods. (C) Multiple sequence alignment of the RRM2 domains of TIA1, PUB1, NAM8 and NGR1 homologs. The figure shows the RNP1 and RNP2 motifs of the domains. Amino acids of the RRM2 (F, R, D, Y, and F) that interact with RNA in the analogous proteins PAB and SXL [74] are highlighted. Conserved positions are shaded in blue. (D) Multiple sequence alignment of the first 70 amino acids of the N-terminus of the U1C homologs. The arrows above the blocks and the lines below indicate the mutations and deletions that were tested in [31]. The color of the arrows indicate whether the position is conserved (orange) or not conserved (green) in the multiple sequence alignment. In the cited study, only the deletions of 30 and 47 amino acids (30AA and 47AA) showed changes in level of interaction with TIA1 [31].

We hypothesized that if the PPTs downstream of the 5′ss are of functional importance in the context of splicing, the presence of these sequences would anti-correlate with the strength of the 5′ss, as they are expected to compensate for weak 5′ss. To assess whether such an anti-correlation exists, we divided all introns into four equally-sized bins of increasing 5′ss strengths. For each bin, we calculated the prevalence of a PPT beginning within the first 20 nt of the intron. Our results demonstrate a clear inverse correlation between 5′ss strength and the presence of a pyrimidine-rich stretch downstream of the 5′ss among all metazoans, excluding *C. elegans* (Figure 6A). Such an anti-correlation was observed in the plant *A. thaliana* as well. These correlations were all highly statistically significant (Supplementary Table 2 in Text S1). However, these anti-correlations were not observed among most fungi and protozoans (Supplementary Figure 3B in Text S1). Thus, these results suggest that among most metazoans and in the plant *A. thaliana*, a pyrimidine-rich stretch downstream of the 5′ss compensates for the presence of a weak 5′ss. This is in agreement with our results pertaining to the *Alu* sequence and with previous molecular studies that found that pyrimidine-rich stretches support the inclusion of weakly defined exons [29],[30],[63].

### TIA proteins are conserved throughout eukaryotic evolution

Given our observation that PPTs downstream of the 5′ss are prevalent throughout evolution, we were next interested in obtaining an evolutionary perspective regarding the TIA proteins, which potentially bind this signal. TIA1 and TIAR proteins are quite similar (81% identity), each contains three RNA-recognition motifs (RRMs) and a glutamine (Q) rich C-terminus [27],[28] and were shown to have redundant activities in splicing [34],[37],[64]. Additionally, we considered two proteins in *S. cerevisiae* that have high similarity to TIA1/TIAR, namely PUB1 and NAM8. Both bind RNA [38],[65],[66] and also have three RRMs. NAM8, which is a constitutive component of the U1 snRNP, binds in a non-specific manner downstream of the 5′ss and affects 5′ss selection [38] and has no counterpart in the mammalian U1 snRNP. As negative controls we included proteins that share high sequence similarity and have similar domain configurations, like the Negative Growth Regulatory protein (NGR1) from *S. cerevisiae* and additional protein families with RNA binding domains (Supplementary Table 3 in Text S1).

Using a combination of hidden Markov models (HMMs) and construction of phylogenetic trees for the candidates (Supplementary Figure 4 in Text S1), we found homologs for TIA1/TIAR in all analyzed metazoans (Figure 5B). In addition, we found that *A. thaliana* and all fungi, except for *S. pombe*, have homologs of PUB1 (Figure 5B). We also found that all fungi, except for *C. neoformans* and *U. maydis*, have homologs of NAM8, whereas its close relative, NGR1, is only present in the group of the *Saccharomycetaceae* (*D. hansenii*, *A. gossypii*, *K. lactis*, *C. glabrata* and *S. cerevisiae*). Finally, we could not detect any clear homologs of TIA1/TIAR, NAM8 or PUB1 in the protozoa *D. discoideum* or *C. parvum*. These results highlight several points. First, among all analyzed organisms excluding protozoa, at least one TIA1/TIAR or PUB1 homolog was found. Second, most organisms for which we demonstrated an anti-correlation between PPT prevalence and 5′ss strength have either TIA1 or TIAR. One exception to this is *C. elegans*, in which there is a TIA1/TIAR homolog, but not a PPT/5′ss anti-correlation, and another is *A. thaliana*, in which an anticorrelation was observed but we found no TIA1/TIAR homologs (see Discussion). Finally, *S. pombe* is an exception among fungi since it lacks any TIA1/TIAR or PUB1 homologs; it also lacks a clear pyrimidine-rich peak downstream of the 5′ss.

### RRM2, responsible for binding U-rich motifs, is the most conserved RRM among TIA homologs

The N-terminal RRM domain in TIA1/TIAR (RRM1) is important for TIA1 activity and enhances the interaction of the Q-rich C-terminal domain with the U1 snRNP [31]. The other two RRMs, RRM2 and RRM3, contact the pre-mRNA, although only RRM2 binds specifically to uridine-rich motifs [31]. RRM2 is the most conserved domain across all homologous proteins (TIA1/TIAR, NAM8 and PUB1), with multiple alignment conservation score of 0.65, as opposed to 0.37 and 0.4 for RRM1 and RRM3, respectively (Figure 5B), and 47% average pairwise identity, as opposed to 37% and 41% for RRM1 and RRM3, respectively. A multiple alignment depicting the conservation of RRM2 across TIA homologs is presented in Figure 6C and alignments for RRM1 and RRM3 are presented in Supplementary Figures 5 and 6 in Text S1, respectively. This conservation underscores the evolutionary importance of the TIA proteins and implies that the mechanism by which TIA homologs bind to RNA has remained conserved throughout evolution.

### The N-terminal region of U1C and the Q-rich C terminus of the TIA proteins are conserved

The recruitment of the U1 snRNP by TIA1 takes place through the interaction of the glutamine-rich (Q-rich) C-terminus of TIA1 with N-terminus of U1C, a protein component of U1 snRNP [31]. We therefore examined U1C conservation. We found U1C homologs in all species analyzed and observed a high degree of conservation among N-terminal regions (Figure 6D) with an average of 69% pairwise similarity in the first 20 positions and much lower conservation levels in downstream residues. In parallel, we examined the extent of conservation of the Q-rich C terminus of the TIA proteins. Although the precise order of amino acids at the C terminus varies, a distinct and statistically significant enrichment was observed in the Q-rich region among the vast majority of TIA1/TIAR/PUB homologs with respect to all other proteins of similar size. Furthermore, no enrichment in Qs was found among relevant controls with high sequence similarity to TIA proteins in other regions (see Supplementary Results in Text S1 for a detailed analysis). Thus, the machinery involved in TIA regulation of splicing is conserved throughout evolution, from the sequences of functional regions of the involved proteins to the binding sites in the pre-mRNA.

## Discussion

This study was motivated by our finding that *Alu* exonization events involving both *Alu* arms occur in only ∼5% of *Alu* exons. Several factors probably limit exonization events across the arms of *Alu* elements. For example, the lengths of exons are known to be constrained with internal exons averaging 145 nucleotides in length. *Alu* exons within right arms average 110 nucleotides in length [67], whereas exons that encompass sequence from both arms tend to be between 200 and 250 nucleotides long. Thus, exonizations occurring from a single arm yield exons that are more optimal in length. However, approximately 20% of human exons are longer than 200 nt [68], strongly contrasting with only 5% of *Alu* exons that contain sequences from both arms.

We hypothesized, and subsequently demonstrated, that the PPT sequence separating the two arms may be involved in limiting exonization across arms. The presence of a PPT enhanced the selection of the 5′ss of the right arm *Alu* exon even in the presence of a stronger splice site downstream. Conversely, in the absence of a PPT sequence between the two splice sites, the stronger downstream site was selected, indicating that in the absence of the PPT, the rules of simple competition apply. In subsequent analyses we were able to determine that the effect of the PPT on the *Alu* 5′ss selection is mediated by TIA1/TIAR proteins. This led us to conduct a bioinformatic analysis in which we examined the machinery involved in TIA regulation across evolution. This machinery, from the binding signal on the pre-mRNA to the sequences of the TIA and U1C proteins, is conserved and, for most metazoans, the presence of a polypyrimidine stretch anti-correlates with 5′ss strength.

Interestingly, our findings may also explain why most exonizations tend to occur predominantly from the right arm of *Alu* elements and not from the left [53]. A previous study showed that exons from within left arms tend to be shorter, depleted in exonic splicing enhancers (ESEs) and enriched in exonic splicing silencers with respect to those from right arms [67]. Here we showed that the presence of a PPT downstream of the right arm *Alu* 5′ss, which is intrinsically embedded in the structure of a typical *Alu* element, enhances the selection of right arm *Alu* exons. Such an effect is not possible in the left arm and this might reduce the potential for *Alu* exonizations from the left arm.

Our study using the *Alu* model system highlights a novel aspect of TIA1/TIAR proteins: These proteins activate a splice site at some distance from their binding site. Previous studies in human systems demonstrated that TIA1 only activates 5′ splice sites immediately followed by U-rich sequences [30],[32], although one study suggested, but did not conclusively prove, that TIA1 may be active from greater distances [62]. In our model system, the PPT was located 18 nt from the 5′ss of the *Alu* exon and the TIA1/TIAR proteins activated its selection. This is similar to the activity of the yeast TIA homolog NAM8 which can activate a 5′ss 46 nt downstream of its binding site [39]. In this respect, our results concur with recent findings, based on depletion of TIA proteins, that demonstrated a correlation between the magnitude of the change in exon skipping and the distance between U-rich motifs and the 5′ss [63]. The function of TIA proteins from a distance may be mediated by other splicing factors or by a formation of pre-mRNA secondary structures that bring together the U-rich sequence and the 5′ss to be activated.

In our experimental system, we focused on the regulative role of the TIA proteins. The reason we focused on these proteins are (1) that the regulation was mediated through the binding to a pyrimidine-rich stretch downstream of the 5′ss, which is a classical mode of regulation of the TIA proteins, and (2) we ruled out PTB, which could potentially also have played a role in this context. However, other splicing factors can bind pyrimidine-rich stretches on the one hand, and play a role in splicing, on the other. Two such proteins are U2AF65 and PUF60: U2AF65 facilitates 3′ splice-site recognition at the early stages of spliceosome assembly, and PUF60 was found to functionally substitute for U2AF65 [69],[70]. Despite the fact that classically these two proteins are mostly known for their involvement in the context 3′ss selection, two considerations could suggest that they might potentially play a role in our system as well: First, the fact that we observed an effect of the PPT when it was distanced up to 18 nucleotides from the 5′ss may suggest that in fact this regulation did not act on the 5′ss but on the 3′ss, since human introns can be as short as 25 nt. In such a scenario, PUF60 and U2AF65 could be involved as factors regulating 3′ss selection. However, we consider this scenario unlikely since the effect we observed increased once the PPT was brought into closer proximity with the 5′ss. Second, it was previously demonstrated that U2AF65 also plays an enhancing regulatory role when binding downstream of the 5′ss [71]. An additional recently discovered protein which might potentially play a role is nSR100, which was shown to bind pyrimidine-rich sequences within alternative exons and in the intronic regions flanking them, and to enhance their recognition [72]. Thus, we cannot rule out that in addition to the TIA proteins, additional factors such as U2AF65 and/or additional factors play a role in *Alu* exonization.

Our bioinformatic analysis provided evidence that our experimental conclusions are applicable to a wide variety of organisms. This analysis showed that the PPT region tends to be located within 20 nt of the beginning of an intron; this is the situation in *Alu* elements. Moreover, this analysis revealed the presence of an inverse correlation between 5′ss strength and prevalence of PPT tracks within metazoan introns. This anticorrelation may be indicative of the functional role of the interaction between these two signals, consistent with previous findings showing that PPTs downstream of the 5′ss support the inclusion of weakly defined exons [29],[30],[32]. It is noteworthy, however, that while our observations establish a correlative relationship between the two signals, it will require experimental analysis in different organisms to establish a cause-effect relationship between the 5′ss and the PPT downstream of it.

Our analysis further demonstrated the high extent of conservation of the TIA proteins and their binding sites on pre-mRNA. For most organisms there was a clear PPT peak downstream of splice sites. In all analyzed eukaryotes, excluding the two protozoa, we found at least one TIA homolog. Moreover, the RRM2 domain, which is responsible for binding U-rich sequences, was particularly conserved and most homologs have retained a glutamine-rich C-terminal region. Finally, the N-terminal domain of U1C, which mediates the recruitment of U1 snRNP by the TIA proteins, was highly conserved among eukaryotes.

Our analysis did, however, show that this machinery may have undergone modifications over the course of evolution. In *S. pombe*, for example, there is no clear PPT downstream of splice sites and we found no PUB1 or TIA1 homolog. This might be related to the extremely short intron length in *S. pombe*, which allows this organism to maintain intron selection without the need for TIA1 or PUB1 proteins. Two additional organisms in which modifications may have occurred are *A. thaliana*, for which no TIA1/TIAR homolog was found, and *C. elegans*, in which no anticorrelation with 5′ss strength existed. One possibility is that in these organisms additional factors compensate for the loss of the factor, or of the signal. Indeed, in plants two related proteins UBP1 and RBP45, can interact with intronic U-rich elements and enhance the recognition of suboptimal splice sites [42]–[44]. This could explain why we still observe a PPT/5′ss anti-correlation in *A. thaliana*. Alternatively, the role of the PPT downstream of the 5′ss, and perhaps also the relationship between the PPT and the 5′ss, may have changed over time. In *C. elegans*, for example, the binding of TIA1/TIAR proteins to the PPT downstream of the 5′ss may occur regardless of the strength of the latter. Alternatively, the PPT downstream of the 5′ss may be an evolutionary ‘fossil’ which has lost its function in *C. elegans*. As in *S. pombe*, such loss of function may be a function of intron length as *C. elegans* introns are considerably shorter than those in other analyzed metazoans [54]. Such loss of function may also be linked with dramatic differences in *C. elegans* splicing compared to other organisms tested in this study, as attested, for example, by the high prevalence of trans-splicing in this organism [73].

A further intriguing result is the balance of power we observe between different splicing signals. Despite the presence of an intervening PPT, a weaker, upstream splice site is only selected as long as the stronger, competing splice site is weaker than a set threshold. Once this threshold is exceeded, the stronger splice site is selected even in the presence of a PPT. The strength of the PPT is yet another factor as also shown by [32]. In this context, we found that in a dataset of 2,296 alternative 5′ss events, in 25.4% and 33.9% of the cases there is a PPT within 25 nt downstream of the proximal and distal 5′ss, respectively. These cases are potential candidates for TIA regulation. Taken together, our findings demonstrate the role of TIA proteins in the specific context of *Alu* exonizations and also in the much wider context of exon selection in organisms from throughout the evolutionary tree.

## Supporting Information

Text S1 Supporting results, supporting methods, supporting figures, and supporting tables.
(7.63 MB DOC)

Dataset S1 Identified homologs for TIA1, TIA1L, ROX8, PUB1, NAM8, NGR1, and CSX1 among the different organisms.
(0.08 MB XLS)
