# Supplementary material for: The Pivotal Roles of TIA Proteins in 5′ Splice-Site Selection of Alu Exons and Across Evolution
Source: PLoS Genet. 2009 Nov 13;5(11):e1000717. doi: 10.1371/journal.pgen.1000717 (PMC2766253; doi:10.1371/journal.pgen.1000717)
Supplement: Text S1 — Supporting results, supporting methods, supporting figures, and supporting tables. (7.63 MB DOC) [file pgen.1000717.s001.doc]

# Supplementary Results

**Analysis of the Q-rich region at the C-terminus of the TIA1/TIAR homolog**s

Homologs in metazoans, except for *C. elegans* and *D. melanogaster*, have a high conservation of glutamine (Q) rich motifs in C-terminal regions. The C-termini of the PUB1 proteins, however, is very variable. We therefore calculated the density of Qs in C-terminal regions of all the TIA-1/TIAR/PUB1 homologs that we found in our analysis. The density was calculated as the number of Qs found in the C-terminal end of the protein (defined as 15% of the length from the C-terminal). We calculated a p-value by comparing with the distribution of the Q-densities calculated for all the proteins for all the species that were of similar size to TIA homologs (between 300 and 500 amino acids).

We observe that almost all TIA1/TIAR/PUB1 homologs have a density of Qs at the C-terminus that is significantly greater than for any other proteins (Supplementary Table 4), with some exceptions:

- One isoform of TIAR in human, which lacks the C-terminal region
- The isoforms in *D. melanogaster*, one case was marginally significant (due to one extra Q)
- The PUB1 homologs in *A. fumigatus*, *Y. lipolytica* (the annotated protein only has two extra amino acids beyond RRM3), *D. hansenii* (the annotated protein only has 25 extra amino acids beyond RRM3) and three proteins in *A. thaliana* (RBP47A, RBP47B, RBP45-like)

As a comparison, we repeated the analysis with the NAM8 homologs (Supplementary Table 5), for which we found no significant enrichment of Qs, except in the *K. lactis* homolog, which is particularly enriched in Q repeats. As a control, we also analyzed two proteins that share high sequence similarity with the TIA1/TIAR/PUB1 homologs: cytoplasmic poly(A)-binding protein in human, PABPC1 (Supplementary Table 6) and the Negative Growth Regulatory protein in *S. cerevisiae*, NGR1 (Supplementary Table 7). The level of enrichment for Qs was not significant for these proteins. Thus, we concluded that although the precise component of amino acids at the N-terminus may vary, the tendency for Q-rich regions at this terminus is highly conserved with few exceptions. Exceptions may be due to redundancy between the activity of the TIA proteins (as in humans, where TIA contains a Q-rich C terminus and TIAR does not) or to changes in the role or mechanism of the proteins.

**Domain conservation between isoforms of TIA homologs**

We were interested in assessing the conservation between the different isoforms of TIA1 [1-3] and TIAR [4,5] (a.k.a. TIAL1) in metazoans (Supplementary Table 8). All TIA1 and TIAR isoforms are highly conserved (Figure S7 especially in the RRM domains (Figures S8, S9, and S10):

- Some of the TIA1 isoforms in human and mouse have an extra linker between RRM1 and RRM2 (Figure S7 This linker is conserved between human and mouse. We used the exonic structure in Ensembl to verify that this extra linker is encoded by a cassette exon in human and mouse (see Figure S11).
- There are some differences in RRM1 sequences: Some of the TIAR isoforms have a long form of RRM1 and some have a short form. The extra sequence is conserved across species (Figure S7). These two different forms were verified by the exonic structure given by Ensembl: The difference is due to alternative splicing at the 3’ end of an exon in both human and mouse (Figure S12). Dog and chicken have the long form of this exon (Figure S12).
- All the *D. melanogaster* isoforms have an extra linker between RRM2 and RRM3 (Figure 2).
- Zebrafish has one extra gene, labelled TIA1L, that has high conservation with TIA1 and TIAR (Figure S7).
- The Q-rich motif at the C-terminus is highly conserved among vertebrates except for a mouse TIA1 isoform, where it is partially missing, and a human TIAR isoform, where it is entirely missing (Figure S7).
- *D. melanogaster* and *C. elegans* homologs have a distinctive repetitive bias at the C-terminus with almost no conservation to the vertebrate C-termini (Figure S7).

Supplementary Methods

**ADAR2 Minigene Insert**

tcataaattggaagaaggaaaggttactcacatcctgatgacatatccattattgagtgacaagaactgtattaattaccctaaacatgtaaaataaatattccagttcaaacttatctttcccttgtggaaaatactagcctctgaaaatgatagactaaaatttaactatatacagctttaaatgttacttttgcaacttttttcctttcagTAACAAAGATGATCAAAAAAGATCCATCTTTCAGAAATCAGAGCGAGGGGGGTTTAGGCTGAAGGAGAATGTCCAGTTTCATCTGTACATCAGCACCTCTCCCTGTGGAGATGCCAGAATCTTCTCACCACATGAGCCAATCCTGGAAGgtatgagacgagattcttcaacaagccagtttctcaagaaaatgttaacagataaaaactaacctgtgttaatatcccttttcctttttttctttttatttttagacttggatgtggatctgataagagtctctataaaagcatggatttttttctttccaattaacttaattagaaaactttaatgaatgaaaaatgctgattgtaattaaccaagtaattagaatggagtgagaatattggattaaaattgtatcagtatttaaatttgggtaataaatatctctaaatcagaggtgtatgtgatcctccatatttctgaggaattctaatttgctaaatgtgtaattaaagtatttatttgcctgatatttcagaagcagataaacactagtgaaaagtgaaaccactattgtgatcatatttttttccatccttctttacatttgaggttagcacttttgttttttagtgttttgtatctacatcccttctcccaagcccttaggcattgtttattccaaatagaacgttttattacattactttccttgtttataaaagcaacaggcatactcactgtggaaaattgctaactatttttaaaatataagaacattaaaatcatccgtattcctgtaacccagaaatactcactatggatgttttaccatactttccatgggtttatttctgcatctgtctagatttattttggaaattttaaaaatatagaggaaaataacatggcaaacacccaagtatccatcacccagaattagcaaatgttagcattttgtcctatttgcttcagtgcatttttaatgatagaaataaatcatttcagttaaagtgaaaaccccatttccccaactcccttggagaccacttttgtgaattttgtttgtgttatgtccttcctactcaatttcatataattttttttttttttttttgagacagGGTCTCGCTCTTACACCCAGGCTGGAGTGCAGTGGTGCAATCATGGCTCACTGCAGCCTCGACCTCCTGGGCTCTTAAGCGATCCTTCCACCTCAACCTTCCAAGGAGCTGGGACTACAGgcatgtaccactatacctggctaatttttatatttttt*acctccactggagaacaatcaaggtttttgctcaaagcttttaagacaactaaagcaaccggatttaggtttgtgaaactctaacagcatccttgtttcactatgacagattctcccttctctctcccctggaccttggccagggaaggtgacagacaactaacacctacttggtgttagtatgtgagcgaagggctttggctgcgtagcctcataatattgtactgacgaggaaagtggggctcagatcatatagtgtattgggggaaggggtgatgagatggatcttggatttgaacccatcagtttgtgttagaacctaagttctctttctatgttataaccctgca*tagagatggggtttcaccatgttgcccaggctggtcttaaactcctgggctcaagcgatccacctgcctcagcctcccaaagtgctgagattacaggtgtgagccaccatacccagcctctcagcttcacatacttattttgtatgtgtattttgtatgtatggcataaacatatgcatttatatgcacagactgtattttaaatttataagctattgttagatgtgcttttcaataaatcaaatagatggtttactacctgtggggttttaactctttttctctcttagAACCAGCAGATAGACACCCAAATCGTAAAGCAAGAGGACAGCTACGGACCAAAATAGAGTCTGGTGAGGGGACGATTCCAGTGCGCTCCAATGCGAGCATCCAAACGTGGGACGGGGTGCTGCAAGGGGAGCGGCTGCTCACCATGTCCTGCAGTGACAAGATTGCACGgtaaggggcgggggctccctgtggccacctccctgcacacaggattcatccatactgtttgccaacctcccttttccacaaccatttgaattttggccccatttcccactcaggtgttccacagtattctttgaaggactgaggttctaaacaggtgctgctgggacccccgtgaggggcagcagcaggacctagacacaggaggctcca

The PPT sequence was replaced with the following sequence that does not contain any known ESRs along it (according to ESEfinder (Cartegni et al, 2003) and ESR Search (http://ast.bioinfo.tau.ac.il/ESR.htm): GCTGCGAGCAACAT

Three inserts were cloned into pBluescript KS+ for in-vitro transcription and pull down assays:

WT:AAGCGATCCTTCCACCTCAACCTTCCAAGGAGCTGGGACTACAGgcatgtaccactatacctggctaatttttatatttttt*acctccactggagaacaatcaagg*

∆PPT:AAGCGATCCTTCCACCTCAACCTTCCAAGGAGCTGGGACTACAGgcatgtaccactatacctggctaa*acctccactggagaacaatcaagg*

rep_PPT:AAGCGATCCTTCCACCTCAACCTTCCAAGGAGCTGGGACTACAGgcatgtaccactatacctggctaagctgcgagcaacat*acctccactggagaacaatcaagg*

Supplementary Tables

| Name of primer | Sequence | Template Plasmid |
| --- | --- | --- |
| ΔPPT-F | P-ACCTCCACTGGAGAACAATCAA | WT |
| ΔPPT-R | P-GCCAGGTATAGTGGTACATGC |  |
| rep_PPT-F | P-AGCAACATACCTCCACTGGAGAACAATCAA | WT |
| rep_PPT-R | P-CGCAGCTTAGCCAGGTATAGTGGTACATGC |  |
| B(3A)_F | AAGCAACCGGATTTAGGTATGTGAAACTCTAACAG | WT |
| B(3A)_R | CTGTTAGAGTTTCACATACCTAAATCCGGTTGCTT |  |
| B(4A)_F | AAGCAACCGGATTTAGGTTAGTGAAACTCTAACAG | WT |
| B(4A)_R | CTGTTAGAGTTTCACTAACCTAAATCCGGTTGCTT |  |
| B(-3C)_F | AAGCAACCGGATTCAGGTTTGTGAAACTCTAACAG | WT |
| B(-3C)_R | CTGTTAGAGTTTCACAAACCTGAATCCGGTTGCTT |  |
| B(3A4A)_F | AAGCAACCGGATTTAGGTAAGTGAAACTCTAACAG | B(3A) |
| B(3A4A)_R | CTGTTAGAGTTTCACTTACCTAAATCCGGTTGCTT |  |
| B(-3C3A4A)_F | AAGCAACCGGATTCAGGTAAGTGAAACTCTAACAG | B(3A4A) |
| B(-3C3A4A)_R | CTGTTAGAGTTTCACTTACCTGAATCCGGTTGCTT |  |
| A(2T)ΔPPT_F | GCTGGGACTACAGGTATGTACCACTATAC | ΔPPT |
| A(2T)ΔPPT_R | GTATAGTGGTACATACCTGTAGTCCCAGC |  |
| 14bpPPT_F | P-TTTTTTTACCTCCACTGGAGAACAATCAA | WT |
| 14bpPPT_R | P-AAAAAAATTAGCCAGGTATAGTGGTACATG |  |
| 9bpPPT_F | P-TTTTTACCTCCACTGGAGAACAATCAA | ΔPPT |
| 9bpPPT_R | P-AAAATTAGCCAGGTATAGTGGTACATG |  |
| 6bpPPT_F | P-TTTACCTCCACTGGAGAACAATCAA | ΔPPT |
| 6bpPPT_R | P-AAATTAGCCAGGTATAGTGGTACATG |  |
| 3bpPPT_F | P-TTACCTCCACTGGAGAACAATCAA | ΔPPT |
| 3bpPPT_R | P-ATTAGCCAGGTATAGTGGTACATG |  |
| Δ5ntPPT_F | P-TAATTTTTATATTTTTTACCTCCACTGG | WT/B(3A)/ B(4A)B(3A4A) |
| Δ5ntPPT_R | P-GTATAGTGGTACATGCCTGTAG |  |
| Δ10ntPPT_F | P-TAATTTTTATATTTTTTACCTCCACTGG | WT/B(3A)/ B(4A)B(3A4A) |
| Δ10ntPPT_R | P-GTGGTACATGCCTGTAGTCC |  |
| U1(5A)_F | CCGAAGATCTCATACATACCTGGCAGGGGAG | U1_WT |
| U1(5A)_R | CTCCCCTGCCAGGTATGTATGAGATCTTCGG |  |
| U1(11A)_F | TCTCATACTTACCTAGCAGGGGAGATACC | U1_WT |
| U1(11A)_R | GGTATCTCCCCTGCTAGGTAAGTATGAGA |  |
| U1(5A11A)_F | CGAAGATCTCATACATACCTAGCAGGGGAGATACC | U1(5A) |
| U1(5A11A)_R | GGTATCTCCCCTGCTAGGTATGTATGAGATCTTCG |  |
| U1(5A6A)_F | CCGAAGATCTCATACAAACCTGGCAGGGGAG | U1(5A) |
| U1(5A6A)_R | CTCCCCTGCCAGGTTTGTATGAGATCTTCGG |  |
| U1(5A6A11A)_F | CCGAAGATCTCATACAAACCTAGCAGGGGAG | U1(5A6A) |
| U1(5A6A11A)_R | CTCCCCTGCTAGGTTTGTATGAGATCTTCGG |  |

**Supplementary Table 1: Primers used throughout the article.** Normal PCR amplifications were done for 30 cycles. Site-directed mutagenesis amplifications were done for 18 cycles.

|  | % PPT introns | Mean PPT length | Position of peak | p value interaction 5'ss-PPT |
| --- | --- | --- | --- | --- |
| *A. thaliana* | 48.42 | 14.12 | 15 | 1.63E-145 |
| *C. parvum* | 25.00 | 13.45 | 69 | 0.721 |
| *D. discoideum* | 35.51 | 19.44 | 89 | 4.58E-07 |
| *S. cerevisiae* | 24.42 | 13.63 | 31 | 0.443 |
| *C. glabrata* | 31.25 | 10.20 | 18 | 0.330 |
| *K. lactis* | 18.90 | 12.79 | 21 | 0.315 |
| *E. gossypi* | 7.80 | 8.53 | 44 | 0.305 |
| *D. hansenii* | 15.03 | 10.56 | 13 | 0.098 |
| *Y. lipolytica* | 8.88 | 11.19 | 71 | 0.037 |
| *N. crassa* | 38.91 | 16.52 | 15 | 4.79E-14 |
| *M. grisea* | 32.55 | 14.02 | 15 | 2.87E-21 |
| *A. fumigatus* | 39.72 | 12.52 | 14 | 0.253 |
| *S. pombe* | 27.17 | 11.45 | 28 | 0.396 |
| *U. maydis* | 21.63 | 12.33 | 77 | 4.33E-06 |
| *C. neoformans* | 38.73 | 13.13 | 14 | 4.56E-13 |
| *C. elegans* | 30.56 | 10.48 | 11 | 9.65E-18 |
| *D. melanogaster* | 17.33 | 10.69 | 36 | 4.91E-15 |
| zebrafish | 24.10 | 12.20 | 16 | 2.29E-102 |
| chicken | 27.62 | 12.36 | 19 | 6.00E-54 |
| dog | 30.13 | 12.92 | 18 | 1.59E-138 |
| mouse | 28.68 | 12.99 | 17 | 8.21E-187 |
| human | 29.79 | 12.90 | 18 | 1.40E-170 |

**Supplementary Table 2:** For each of the 22 organisms, the percentage of introns with a PPT, the mean PPT length, the peak position (within the first 100 nt), and the χ^2^ p-value for the interaction between the 5’ss and the PPT following division into 4 bins (as in Figure 5) is presented.

| **Protein subset** | **Families used for building the HMMs** | |
| --- | --- | --- |
| **SR proteins** | ASF/SF2, SRp30C | RY1 |
|  | SRp20, 9G8 | SRm300 |
|  | SRp40, SRp55, SRp75 | Topo I-B |
|  | SC35, SRp46 | p54, SRp86 |
| **SR-related proteins** | U2AF35 | TRA2 |
|  | U2AF65 | RNPS1 |
|  | MUD2 | U1-70K |
|  | NPL3 |  |
| **hnRNPs** | Musashi | hnRNP-I |
|  | hnRNP-A | hnRNP-K |
|  | hnRNP-C | hnRNP-L |
|  | hnRNP-D | hnRNP-M |
|  | hnRNP-F-H | hnRNP-R |
|  | hnRNP-E | hnRNP-K |
|  | hnRNP-G |  |
| **Other proteins** | CUG | FUSE |
|  | ELAV | TIA1, TIAR, PUB1 |
|  | NAM8, NGR1 | PAB1/PABPC1 |
|  | U1A | U2B’’ |
|  | U1C | U2A |

**Supplementary Table 3:** List of protein families used to create the HMMs for the homolog search. We built independent HMMs for each of the protein domains or a single HMM for the whole protein when no known structural domain was identified (e.g. SRm300). Proteins with high similarity were classified into the same family (e.g. TIA1, TIAR and PUB1).

| **species** | **gene** | **protein** | **Q-density at the C-terminus (15%)** | **p-value** |  |
| --- | --- | --- | --- | --- | --- |
| *H sapiens* | TIA1 | ENSG00000116001|ENST00000282574 | 0.175 | 5.52E-05 | * |
|  |  | ENSG00000116001|ENST00000361692 | 0.178 | 3.85E-05 | * |
|  | TIAL1 | ENSG00000151923|ENST00000369088 | 0.044 | 0.4752803 |  |
|  |  | ENSG00000151923|ENST00000369092 | 0.178 | 3.85E-05 | * |
|  |  | ENSG00000151923|ENST00000369093 | 0.172 | 7.86E-05 | * |
| *M musculus* | TIA1 | ENSMUSG00000071337|ENSMUSP00000093424 | 0.16 | 0.000300931 | * |
|  |  | ENSMUSG00000071337|ENSMUSP00000093425 | 0.157 | 0.000413419 | * |
|  |  | ENSMUSG00000071337|ENSMUSP00000093426 | 0.14 | 0.002186295 | * |
|  |  | ENSMUSG00000071337|ENSMUSP00000109341 | 0.142 | 0.001818546 | * |
|  |  | ENSMUSG00000071337|ENSMUSP00000109342 | 0.16 | 0.000300931 | * |
|  | TIAR | ENSMUSG00000030846|ENSMUSP00000033135 | 0.16 | 0.000300931 | * |
|  |  | ENSMUSG00000030846|ENSMUSP00000101833 | 0.155 | 0.000508884 | * |
| *C familiaris* | TIA1 | ENSCAFG00000003341|ENSCAFP00000004967 | 0.155 | 0.000508884 | * |
|  | TIAL1 | ENSCAFG00000012171|ENSCAFP00000017967 | 0.172 | 7.86E-05 | * |
| *G gallus* | TIA1 | ENSGALG00000013879|ENSGALP00000022466 | 0.181 | 2.67E-05 | * |
|  | TIAL1 | ENSGALG00000009427|ENSGALP00000015333 | 0.189 | 9.64E-06 | * |
|  |  | ENSGALG00000009427|ENSGALP00000029088 | 0.189 | 9.64E-06 | * |
| *D rerio* | TIA1 | ENSDARG00000052536|ENSDARP00000068889 | 0.105 | 0.03336324 | * |
|  | TIA1L | ENSDARG00000026476|ENSDARP00000018009 | 0.235 | 1.02E-08 | * |
|  | TIAL1 | ENSDARG00000009525|ENSDARP00000020234 | 0.2 | 2.19E-06 | * |
| *D melanogaster* | ROX8 | CG5422|Rox8-RB|CG5422-RB | 0.086 | 0.09996863 |  |
|  |  | CG5422|Rox8-RC|CG5422-RC | 0.086 | 0.09996863 |  |
|  |  | CG5422|Rox8-RD|CG5422-RD | 0.1 | 0.04567623 | * |
|  |  | CG5422|Rox8-RF|CG5422-RF | 0.086 | 0.09996863 |  |
| *C elegans* | TIA1 | C18A3.5|C18A3.5a | 0.278 | 3.50E-12 | * |
|  |  | C18A3.5|C18A3.5b | 0.285 | 8.27E-13 | * |
|  | TIAR | Y46G5A.13|Y46G5A.13 | 0.123 | 0.00923019 | * |
| *C neoformans* | PUB1 | cneo_JEC21_TIGR:CND05240 | 0.184 | 1.83E-05 | * |
| *U maydis* | PUB1 | umay_BRD:UM01182.1 | 0.173 | 7.00E-05 | * |
| *A fumigatus* | PUB1 | Afu1g12000 | 0.054 | 0.3622639 |  |
| *M grisea* | PUB1 | MGG_06400 | 0.178 | 3.85E-05 | * |
| *N crassa* | PUB1 | contig_3.474_1 | 0.137 | 0.002865147 | * |
| *Y lipolytica* | PUB1 | ylip_GENO:CAG81845.1 | 0.033 | 0.3984158 |  |
| *D hansenii* | PUB1 | dhan_GENO:CAG84729.1 | 0.057 | 0.330136 |  |
| *A gossypii* | PUB1 | agos_GBK:AGR390C | 0.107 | 0.02927254 | * |
| *K lactis* | PUB1 | klac_GENO:CAG99432.1 | 0.267 | 3.11E-11 | * |
| *C glabrata* | PUB1 | cgla_GENO:CAG60192.1 | 0.112 | 0.02083506 | * |
| *S cerevisiae* | PUB1 | YNL016W.1 | 0.343 | 1.11E-18 | * |
| *A thaliana* | RBP47A | AT1G49600.1-TAIR|AT1G49600.1-P|AT1G49600 | 0.06 | 0.2992145 |  |
|  | RBP47B | AT3G19130.1-TAIR|AT3G19130.1-P|AT3G19130 | 0.046 | 0.4522079 |  |
|  | RBP47C | AT1G47490.1-TAIR|AT1G47490.1-P|AT1G47490 | 0.109 | 0.02560735 | * |
|  | RBP47C2 | AT1G47500.1-TAIR|AT1G47500.1-P|AT1G47500 | 0.107 | 0.02927254 | * |
|  | UBP1A | AT1G54080.1-TAIR|AT1G54080.1-P|AT1G54080 | 0.126 | 0.007275292 | * |
|  | UBP1B | AT1G17370.1-TAIR|AT1G17370.1-P|AT1G17370 | 0.145 | 0.001371518 | * |
|  | UBP1C | AT3G14100.1-TAIR|AT3G14100.1-P|AT3G14100 | 0.14 | 0.002186295 | * |
|  | RBP45A | AT5G54900.1-TAIR|AT5G54900.1-P|AT5G54900 | 0.189 | 9.64E-06 | * |
|  | RBP45B | AT1G11650.2-TAIR|AT1G11650.2-P|AT1G11650 | 0.25 | 7.49E-10 | * |
|  | RBP45C | AT4G27000.1-TAIR|AT4G27000.1-P|AT4G27000 | 0.209 | 6.06E-07 | * |
|  | RBP45-like | AT5G19350.1-TAIR|AT5G19350.1-P|AT5G19350 | 0.063 | 0.2696789 |  |

**Supplementary Table 4:** Density of glutamine (Q) repeats at the C-terminus of each of the TIA1/TIAR isoforms. The density was calculated as described in the text. The p-value was calculated by comparison with the distribution of the densities for Q for the proteomes of all the species considered. P-values less than 0.05 are indicated with an asterisk.

| **Species** | **gene** | **protein** | **Q-density at the C-terminus (15%)** | **p-value** |  |
| --- | --- | --- | --- | --- | --- |
| *S pombe* | NAM8 | spom_SANG:SPBPJ758.01 | 0.071 | 0.1987448 |  |
| *A fumigatus* | NAM8 | Afu7g02230 | 0.048 | 0.4292958 |  |
| *M grisea* | NAM8 | MGG_08741 | 0.048 | 0.4292958 |  |
| *N crassa* | NAM8 | contig_3.27_6 | 0.037 | 0.4438224 |  |
| *Y lipolytica* | NAM8 | ylip_GENO:CAG80611.1 | 0.04 | 0.4784032 |  |
| *D hansenii* | NAM8 | dhan_GENO:CAG89760.1 | 0.014 | 0.2091911 |  |
| *A gossypii* | NAM8 | agos_GBK:ADR307W | 0.083 | 0.1161203 |  |
| *K lactis* | NAM8 | klac_GENO:CAG98456.1 | 0.136 | 0.003130527 | * |
| *C glabrata* | NAM8 | cgla_GENO:CAG59820.1 | 0.048 | 0.4292958 |  |
| *S cerevisiae* | NAM8 | YHR086W.1 | 0.064 | 0.2601687 |  |

**Supplementary Table 5**: Densities of glutamines in NAM8 homologs. Significant p-values are indicated with an asterisk.

| **Species** | **gene** | **Protein** | **Q-density at the C-terminus (15%)** | **p-value** |
| --- | --- | --- | --- | --- |
| *H sapiens* | PABPC1L | H_sapiens|PABPC1L|20|ENSG00000101104|ENST00000217074 | 0.081 | 0.1278685 |
| *M muculus* | PABPC4 | M_musculus|PABPC4|ENSMUSG00000011257|ENSMUSP00000091744 | 0.068 | 0.2239293 |
| *C familiaris* | PABPC5 | C_familiaris|PABPC5|ENSCAFP00000025717 | 0.025 | 0.3121453 |
| *G gallus* | PABPC1 | G_gallus|PABPC1|ENSGALP00000023288 | 0.082 | 0.121895 |
| *C elegans* | PAB1 | C_elegans|PAB1|I|Y106G6H.2|Y106G6H.2c | 0.08 | 0.1340425 |

**Supplementary Table 6**: Densitities of glutamines in PAB1 homologs in metazoans.

| **Species** | **gene** | **Protein** | **Q-density at the C-terminus (15%)** | **p-value** |  |
| --- | --- | --- | --- | --- | --- |
| *S pombe* | NGR1 | spom_SANG:SPAC17A2.09c | 0.063 | 0.2686934 |  |
| *D hansenii* | NGR1 | dhan_GENO:CAG88784.1 | 0.104 | 0.03557877 | * |
| *A gossypii* | NGR1 | agos_GBK:AAR151W | 0.125 | 0.007881992 | * |
| *K lactis* | NGR1 | klac_GENO:CAH01296.1 | 0.037 | 0.4438224 |  |
| *C glabrata* | NGR1 | cgla_GENO:CAG61473.1 | 0.066 | 0.2416803 |  |
| *S cerevisiae* | NGR1 | YBR212W.1 | 0.02 | 0.2627193 |  |

**Supplementary Table 7**: Densitities of glutamines in NGR1 homologs in fungi.

| Species | Gene Name | Gene ID | Isoform ID |
| --- | --- | --- | --- |
| *H sapiens* | TIA1 | ENSG00000116001 | ENST00000282574  ENST00000361692 |
|  | TIAL1 | ENSG00000151923 | ENST00000369088  ENST00000369092  ENST00000369093 |
| *M musculus* | TIA1 | ENSMUSG00000071337 | ENSMUSP00000093424  ENSMUSP00000093425  ENSMUSP00000093426  ENSMUSP00000109341  ENSMUSP00000109342 |
|  | TIAR | ENSMUSG00000030846 | ENSMUSP00000033135  ENSMUSP00000101833 |
| *C familiaris* | TIA1 | ENSCAFG00000003341 | ENSCAFP00000004967 |
|  | TIAR | ENSCAFG00000012171 | ENSCAFP00000017967 |
| *G gallus* | TIAL1 | ENSGALG00000009427 | ENSGALP00000015333  ENSGALP00000029088 |
|  | TIA1 | ENSGALG00000013879 | ENSGALP00000022466 |
| *D rerio* | TIA1 | ENSDARG00000052536 | ENSDARP00000068889 |
|  | TIAL1 | ENSDARG00000009525 | ENSDARP00000020234 |
|  | TIA1L | ENSDARG00000026476 | ENSDARP00000018009 |
| *D melanogaster* | ROX8 | Rox8|CG5422 | Rox8-RB|CG5422-RB  Rox8-RC|CG5422-RC  Rox8-RD|CG5422-RD  Rox8-RF|CG5422-RF |
| *C elegans* | TIA1 | C18A3.5 | C18A3.5a  C18A3.5b |
|  | TIAR | Y46G5A.13 | Y46G5A.13 |

**Supplementary Table 8**: Known isoforms for each of the TIA1 and and homologs TIAR (a.k.a TIAL or TIAL1) and TIA1L (in *D. rerio*) and ROX8 (in *D. melanogaster*).

Supplementary Figures


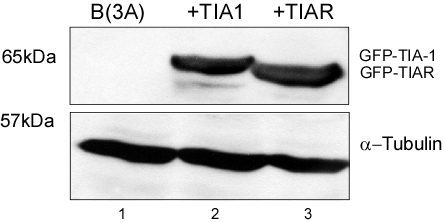


**Figure 1:** Western blotting of 293T transfected cells with B(3A) ADAR2 mutant (lane 1) and TIA1/TIAR (lane 2 and 3) using anti-GFP and anti-tubulin antibodies.


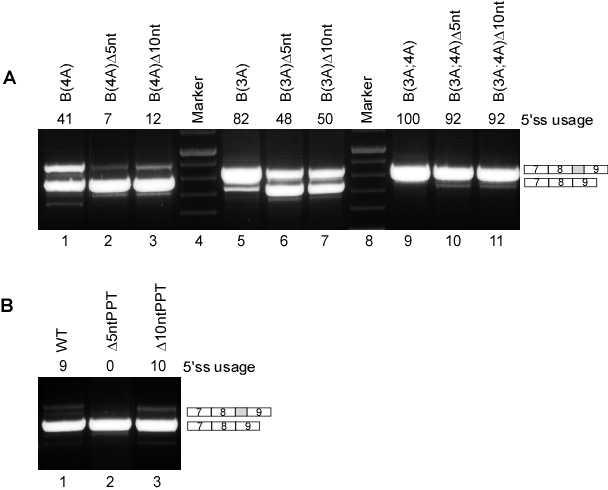


**Figure 2:** Shortening the distance between 5'ssA and the PPT effects 5'ss selection. (**A**) Lanes 1-3, lanes 5-7 and lanes 9-11 - splicing products of mutations 5’ssB(4A), 5’ssB(3A) and 5’ssB(3A;4A) respectively combined with a deletion of 5nt or 10nt between 5'ssA and the PPT; lanes 4 and 8; DNA marker. (**B**) Lane 1; splicing products of wild-type ADAR2; lanes 2-3; splicing products of wild-type ADAR2 combined with a deletion of 5nt or 10nt between 5'ssA and the PPT.


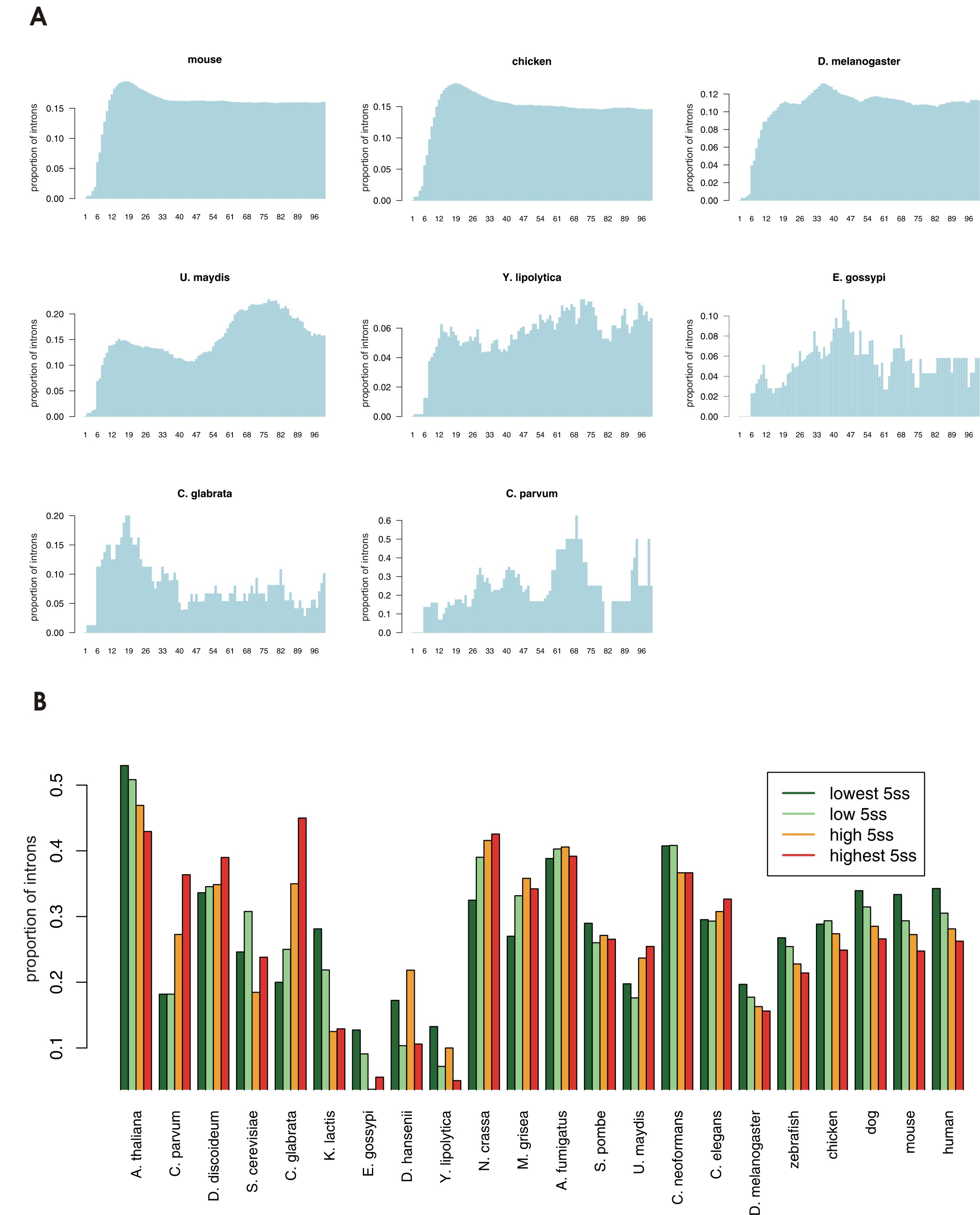


**Figure 3: (A)** Prevalence of PPTs downstream of 5’ss among different organisms (as in Figure 5). **(B)** Prevalence of PPTs among the 22 organisms, divided into four bins based on increasing 5’ss strength.

**Figure 4:** Maximum parsimony (MP) tree of the RRM2 from the TIA1, PUB1, NAM8 and NGR1 homologs (TIAR homologs are omitted as their sequence is redundant with TIA1) using the Close-Neighbour-Interchange algorithm with search level 3. The initial trees were obtained with random addition of sequences using 10 replicates. Multiple alignments were built using t-coffee [6] and phylogenetic analyses were performed with MEGA4 [7].


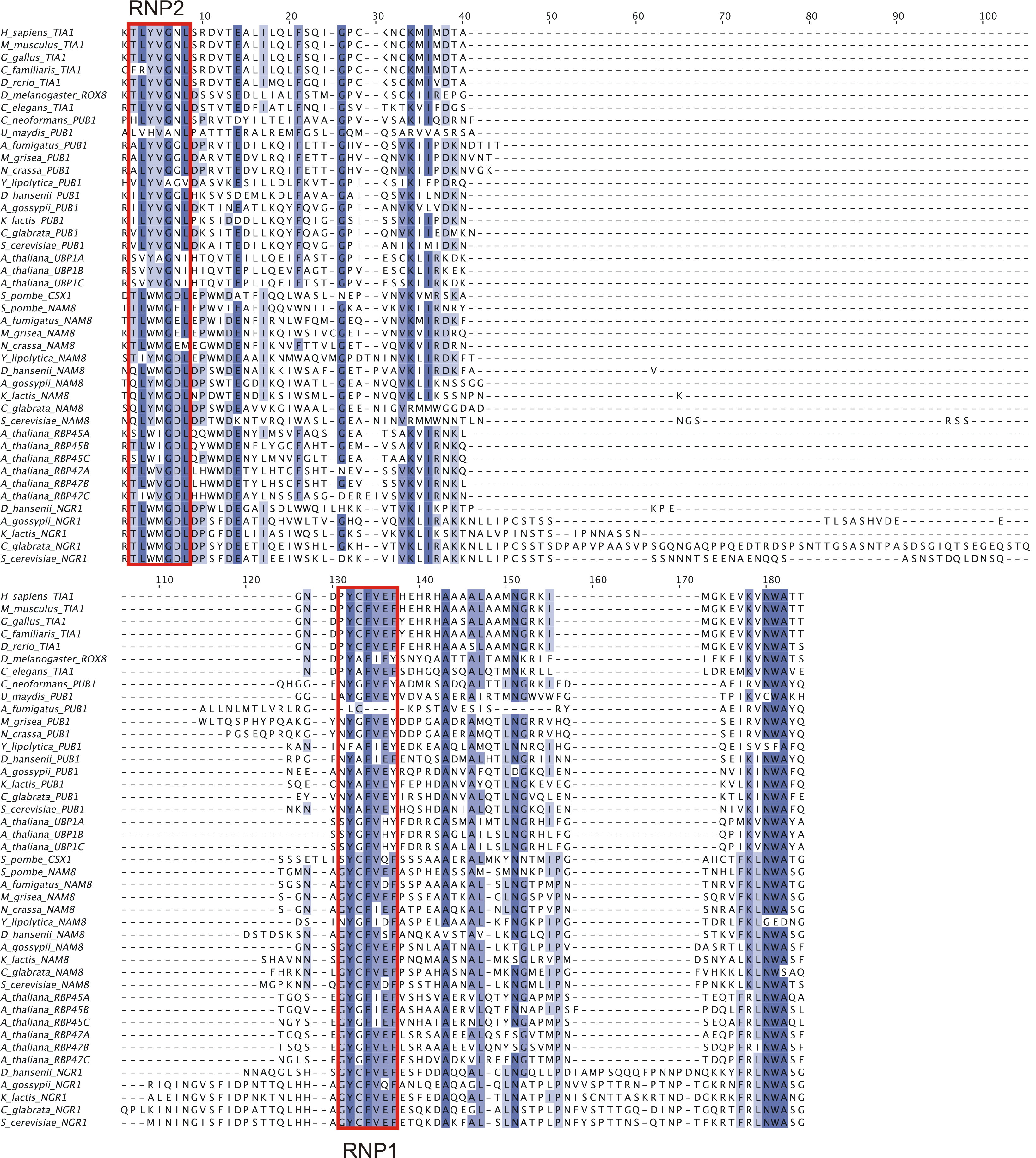


**Figure 5.** Multiple sequence alignment of the RRM1 domain of TIA1, PUB1, NAM8 and NGR1 homologs. TIAR homologs are omitted as their sequence is redundant. Conserved positions are shaded in blue. The RNP1 and RNP2 motifs are highlighted.


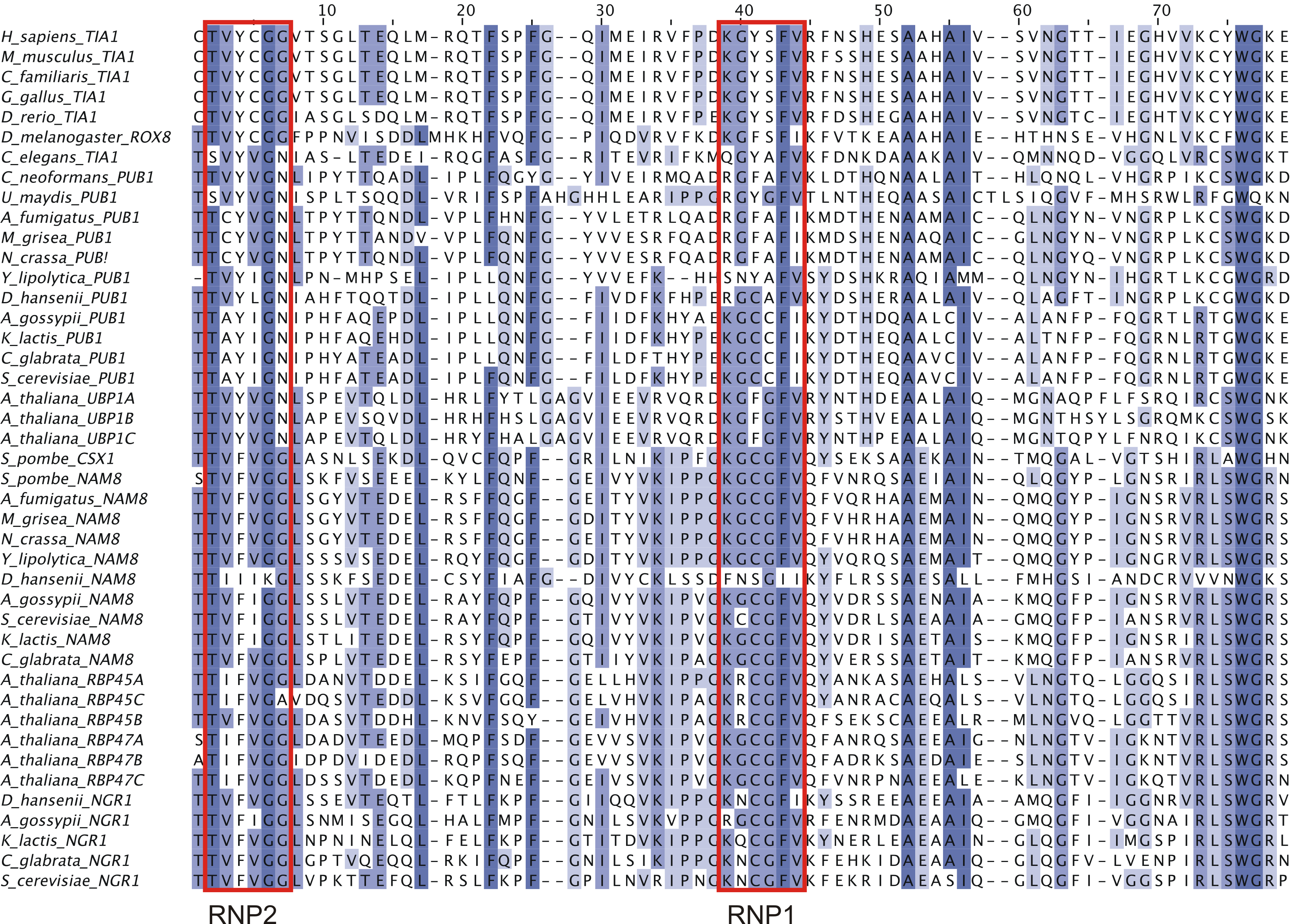


**Figure 6.** Multiple sequence alignment of the RRM3 domain of TIA1, PUB1, NAM8 and NGR1 homologs. TIAR homologs are omitted as their sequence is redundant. Conserved positions are shaded in blue. The RNP1 and RNP2 motifs are highlighted.


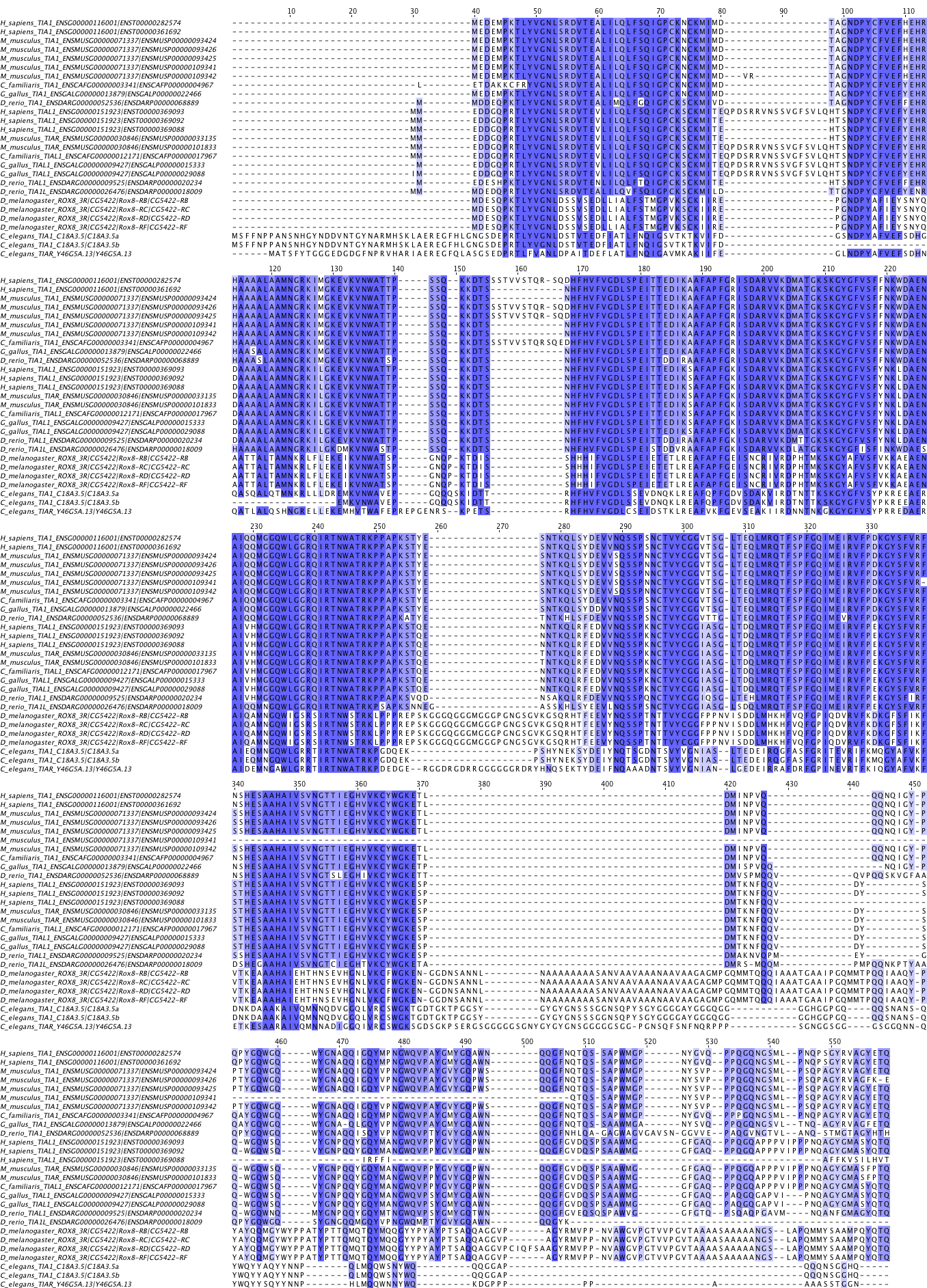


**Figure 7**. Multiple sequence alignment of the TIA1 and TIAR isoforms.


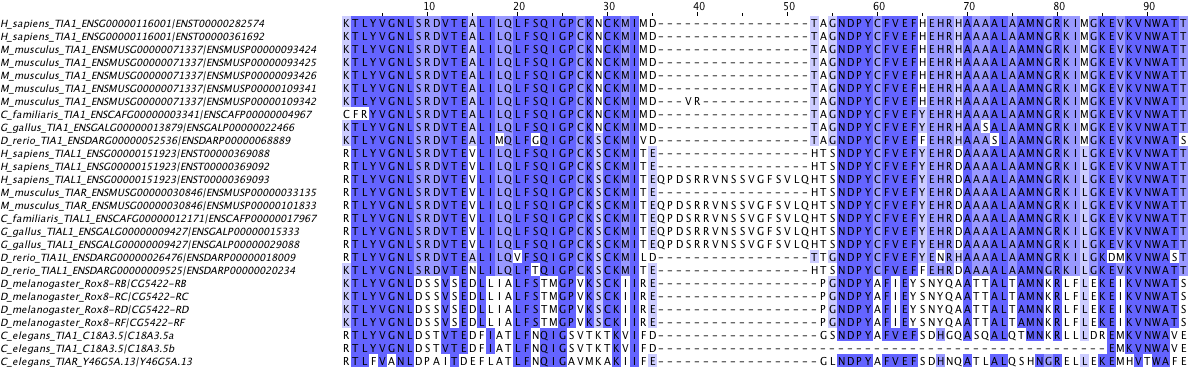
**Figure 8**. Multiple alignment of the RRM1 for the TIA1 and TIAL/TIAR isoforms


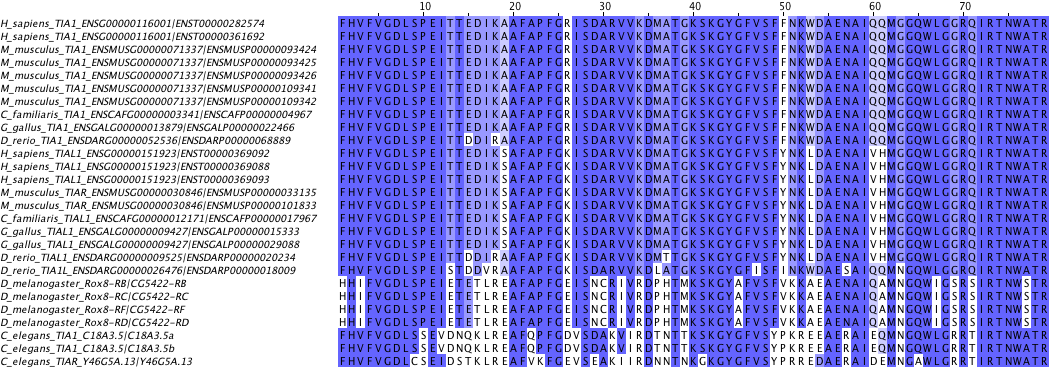


**Figure 9**. Multiple alignment of the RRM2 for the TIA1 and TIAR isoforms


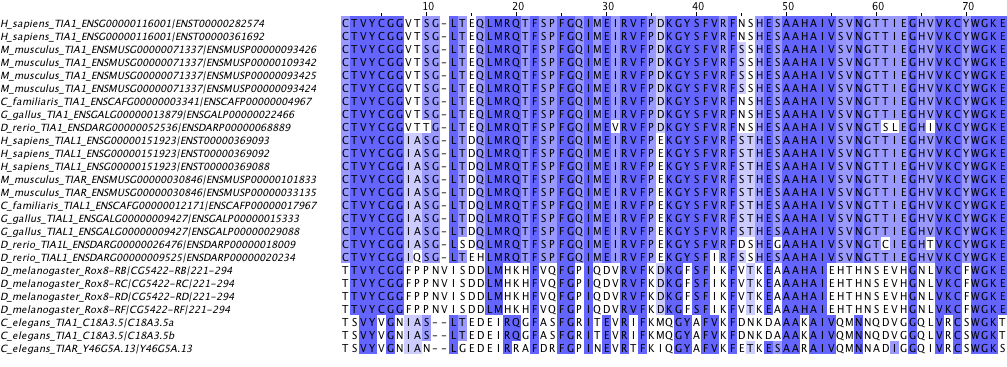


**Figure 10**. Multiple alignment of the RRM3 for the TIA1 and TIAR isoforms

**Figure 11:** We extracted from Ensembl the annotation for the TIA1 gene for human and mouse, and exported the image of the “splice variants view” with protein annotations. The figure shows the exon-intron structure and protein organizations of both genes. The annotation shows that both human and mouse TIA1 have a cassette exon that lies between RRM1 and RRM2.

**Figure 12:** Ensembl annotations were extracted as above for human, mouse, chicken and dog. The figure shows that there is an alternative exon in human and mouse that makes an isoform of TIAR with a longer RRM1. Moreover, dog and chicken contain the long form of this exon.

**References**

1. Kawakami A, Tian Q, Streuli M, Poe M, Edelhoff S, et al. (1994) Intron-exon organization and chromosomal localization of the human TIA-1 gene. J Immunol 152: 4937-4945.

2. Lowin B, French L, Martinou JC, Tschopp J (1996) Expression of the CTL-associated protein TIA-1 during murine embryogenesis. J Immunol 157: 1448-1454.

3. Tian Q, Streuli M, Saito H, Schlossman SF, Anderson P (1991) A polyadenylate binding protein localized to the granules of cytolytic lymphocytes induces DNA fragmentation in target cells. Cell 67: 629-639.

4. Beck AR, Medley QG, O'Brien S, Anderson P, Streuli M (1996) Structure, tissue distribution and genomic organization of the murine RRM-type RNA binding proteins TIA-1 and TIAR. Nucleic Acids Res 24: 3829-3835.

5. Kawakami A, Tian Q, Duan X, Streuli M, Schlossman SF, et al. (1992) Identification and functional characterization of a TIA-1-related nucleolysin. Proc Natl Acad Sci U S A 89: 8681-8685.

6. Notredame C, Higgins DG, Heringa J (2000) T-Coffee: A novel method for fast and accurate multiple sequence alignment. J Mol Biol 302: 205-217.

7. Tamura K, Dudley J, Nei M, Kumar S (2007) MEGA4: Molecular Evolutionary Genetics Analysis (MEGA) software version 4.0. Mol Biol Evol 24: 1596-1599.
